# Characterization of anticancer therapy–induced microvascular dysfunction in patients with breast cancer supports targeted intervention

**DOI:** 10.1172/jci.insight.194316

**Published:** 2025-09-30

**Authors:** Janée D. Terwoord, Laura E. Norwood Toro, Shelby N. Hader, Stephen T. Hammond, Joseph C. Hockenberry, Jasmine Linn, Ibrahim Y. Vazirabad, Amanda L. Kong, Alison J. Kriegel, Ziqing Liu, Riikka M. Kivelä, Gillian Murtagh, David D. Gutterman, Andreas M. Beyer

**Affiliations:** 1 Cardiovascular Center, Department of Medicine, Medical College of Wisconsin, Milwaukee, Wisconsin, USA.; 2 Biomedical Sciences Department, Rocky Vista University, Ivins, Utah, USA.; 3 Department of Physiology, Medical College of Wisconsin, Milwaukee, Wisconsin, USA.; 4 Department of Surgery, Froedtert and Medical College of Wisconsin, Milwaukee, Wisconsin, USA.; 5 Wihuri Research Institute and Stem Cells and Metabolism Research Program, Faculty of Medicine, University of Helsinki, Helsinki, Finland.; 6 Faculty of Sport and Health Sciences, University of Jyväskylä, Jyväskylä, Finland.; 7 Core Diagnostics, Abbott Laboratories, Abbott Park, Illinois, USA.

**Keywords:** Cardiology, Oncology, Vascular biology, Breast cancer, Endothelial cells, Microcirculation

## Abstract

**BACKGROUND:**

Cardiotoxicity is a major complication of anticancer therapy (CTx); however, the effect of CTx on the microcirculation is not well defined. This study evaluated the effect of CTx on microvascular function in patients with breast cancer (PwBC).

**METHODS:**

Endothelial function and angiogenic potential were assessed in arterioles and adipose biopsies obtained from PwBC undergoing CTx (longitudinal and cross-sectional) and in healthy arterioles exposed to doxorubicin (Dox), trastuzumab (TZM), or paclitaxel (PTX) ex vivo. VEGF-B protein was used to test feasibility of targeted intervention.

**RESULTS:**

PwBC treated with Dox and/or TZM developed profound microvascular endothelial dysfunction that persisted for ≥ 9 months after treatment cessation. Angiogenic potential was reduced during CTx and recovered within 1 month. Gene expression related to angiogenesis and inflammation changed over the course of clinical treatment. Adipose arterioles from healthy donors developed endothelial dysfunction when exposed to Dox or TZM ex vivo. PTX, which poses minimal cardiovascular risk, had no effect on vasomotor function. Ex vivo exposure to Dox or PTX suppressed angiogenic potential, whereas TZM had no effect. VEGF-B protein preserved endothelial function in arterioles exposed to Dox or TZM ex vivo.

**CONCLUSION:**

PwBC undergoing treatment with Dox and/or TZM develop prolonged microvascular endothelial dysfunction that is recapitulated in healthy arterioles exposed to Dox or TZM ex vivo. Targeted intervention with VEGF-B protects against direct Dox- or TZM-induced vascular toxicity in human arterioles ex vivo.

**FUNDING:**

NIH, American Heart Association, WeCare Foundation, Medical College of Wisconsin, Advancing a Healthier Wisconsin, Jenny and Antti Wihuri Foundation.

## Abstract 2

<span>Chemotherapy-induced vascular toxicity is important because it can raise the risk or cause for heart disease and stroke in cancer survivors—even years after treatment.</span>

## Graphical abstract

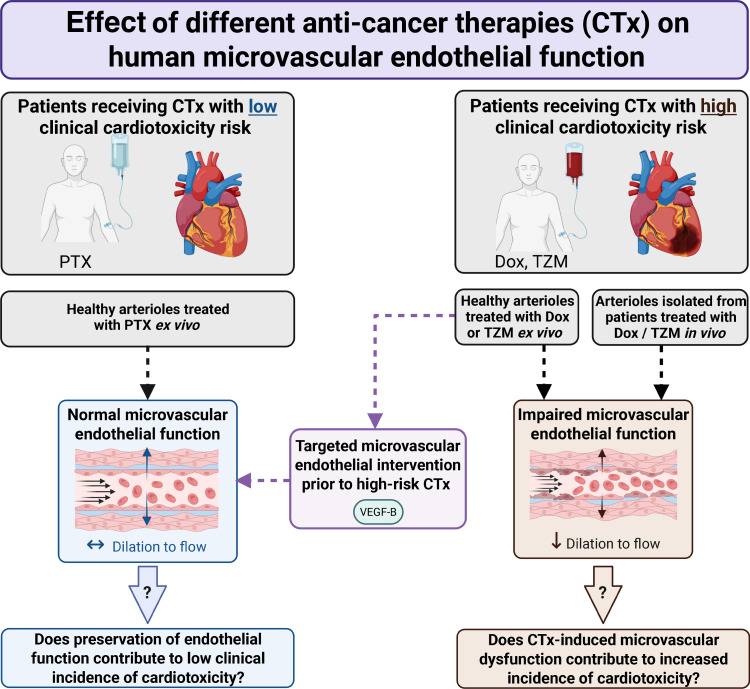

## Introduction

Cancer survivorship continues to climb with earlier detection and improved efficacy of anticancer therapy (CTx) (1); however, cardiotoxicity remains a major limitation of CTx, with acute and long-term consequences for patients and cancer survivors (2). Breast cancer (BC) survivors have elevated risk of cardiovascular disease compared with peers without cancer: the risk of mortality from heart disease is 2- to 6-fold greater in patients with BC depending upon age at diagnosis (3). Indeed, cardiovascular disease is the leading noncancer cause of mortality among patients with BC (4). Thus, new strategies are urgently needed to prevent and treat CTx-induced cardiovascular toxicity.

Clinical use of several CTx agents commonly prescribed to treat BC is limited by cardiotoxicity (5). Anthracyclines, including doxorubicin (Dox), induce dose-dependent, irreversible reductions in ejection fraction (2), while targeted anti-HER2 molecular therapies, such as trastuzumab (TZM), induce acute cardiotoxicity that is often reversible (2). In contrast, microtubule inhibitors, such as paclitaxel (PTX), are not generally associated with cardiotoxicity, as they have minimal effect on cardiac contractile function despite an increased risk of arrhythmia (5, 6). Understanding mechanistic differences between CTx agents with high versus low risk of cardiotoxicity will inform opportunities to improve clinical care.

Although cardio-oncology research has largely focused on the direct effect of CTx agents on cardiomyocytes, vascular endothelial dysfunction has emerged as another putative factor that may contribute to cardiotoxicity (5, 7). Microvascular function is crucial for ensuring perfusion of vital organs, including the heart, and endothelial dysfunction could promote cardiotoxicity via a reduction in myocardial perfusion, increased afterload on the heart, and/or altered paracellular signaling between endothelial cells (ECs) and cardiomyocytes. In patients with BC, impaired coronary microvascular function is associated with heightened risk of future cardiovascular events (8). The microvascular endothelium also regulates angiogenesis, which is disrupted as an on- or off-target effect of some CTx agents (9, 10). Despite the importance of the microcirculation to cardiovascular health, little is known about the effect of CTx on human microvascular function.

ECs are directly exposed to circulating CTx agents, and we previously reported endothelial dysfunction in human coronary arterioles exposed to Dox ex vivo (11). Dox-induced coronary microvascular damage has been reported in preclinical models (12, 13), which raises the possibility that microvascular toxicity may contribute to cardiac dysfunction. Recent cross-sectional studies reveal microvascular dysfunction in patients with cancer undergoing chemotherapy (14, 15); however, the direct effect of CTx on human microvascular function has not been investigated in a longitudinal manner.

Vascular endothelial growth factors (VEGF-A and VEGF-B) and their receptors (VEGFRs) regulate the endothelium and play a role in physiological and tumor angiogenesis. Tumor overexpression of VEGF-A promotes tumor vascularization primarily via VEGFR-2 signaling; thus, most antiangiogenic CTx agents target VEGF-A or VEGFR-2 (16). In contrast, VEGF-B, which acts on VEGFR-1 and the coreceptor neuropilin receptor-1 (NRP1), has minimal effects on angiogenesis in most tissues (17, 18). Rather, VEGF-B acts as a protective factor that promotes EC survival during stress (18, 19). VEGF-B is abundantly expressed in the heart, where it maintains the vasculature and preserves vessel density (20, 21). VEGF-B overexpression has shown promise in preventing Dox-induced cardiotoxicity in mice (13); however, it is unclear whether VEGF-B protects the human microcirculation from Dox- or TZM-induced damage.

The present study was designed to provide a comprehensive assessment of the effects of 3 commonly used BC therapies on key aspects of microvascular endothelial health, including vasomotor function, angiogenesis, and gene expression. The goals were to (a) test the hypothesis that the cardiotoxic CTx agents Dox and TZM induce microvascular endothelial dysfunction and (b) provide molecular insight and proof-of-principle data regarding the potential utility of targeted VEGF-B therapy to counteract CTx-induced vascular toxicity.

## Results

### CTx induces microvascular dysfunction and suppresses angiogenic potential in patients with BC.

Twenty-two patients diagnosed with BC participated in a longitudinal study to evaluate the effects of CTx on the vasculature with measurements before, during, and approximately 1 month after the final dose of Dox or anti-HER2 therapy received during standard-of-care treatment. During periods of high COVID-19 infection levels, mid-CTx study visits were canceled, as these visits were not required for patient care. Longitudinal study participant characteristics at the baseline (pre-CTx) visit and CTx information are provided in Table 1. Prior to CTx, 18 patients (82%) had zero cardiovascular risk factors, 2 patients (9%) had 1 cardiovascular risk factor, and 2 patients (9%) had 2 cardiovascular risk factors. Patients underwent treatment with Dox (59% of patients), anti-HER2 monoclonal antibody therapy (e.g., TZM) (27%), or combined Dox plus anti-HER2 therapy (14%).

**Figure 1:**
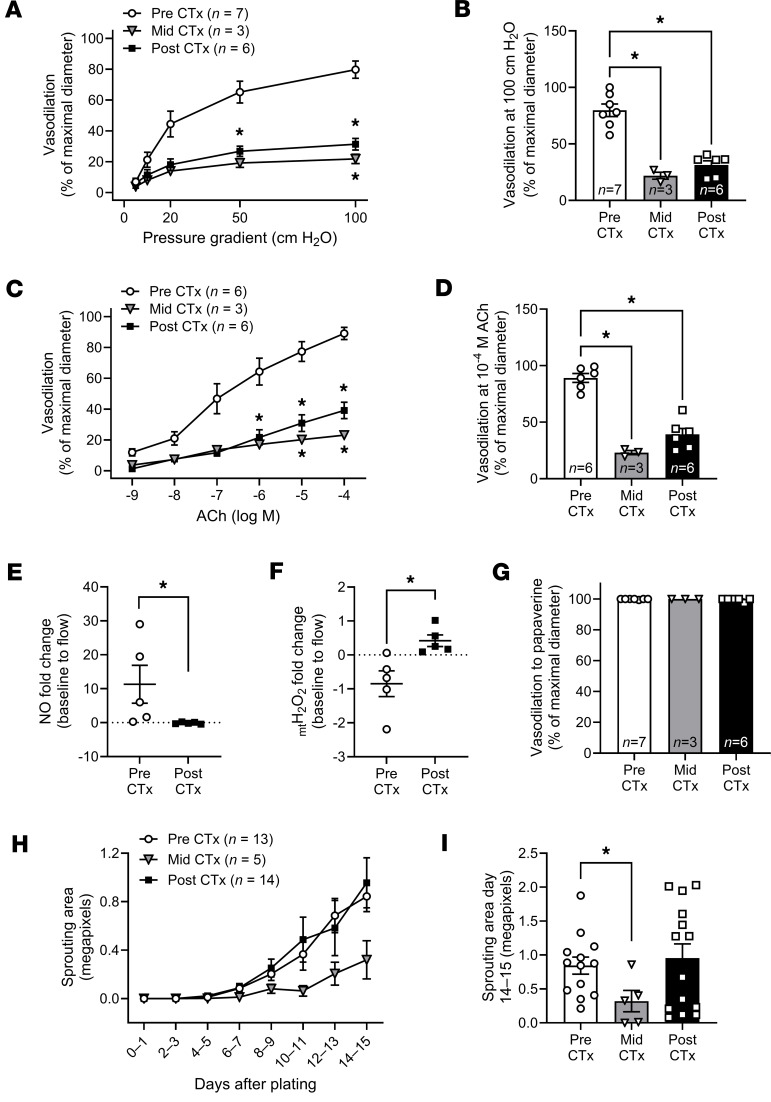
CTx induces microvascular dysfunction and suppresses angiogenic potential in patients with BC. (**A**–**D**) In a longitudinal study of patients with BC, endothelium-dependent dilation to flow (**A** and **B**) and acetylcholine (ACh) (**C** and **D**) were reduced in adipose arterioles isolated during and 1 month after CTx. (**E** and **F**) One-month after CTx, flow-induced nitric oxide (NO) production was suppressed (**E**) while mitochondrial hydrogen peroxide (_mt_H_2_O_2_) production was elevated (**F**) in patient arterioles (measured via immunofluorescent probes). (**G**) Endothelium-independent dilation to papaverine was preserved. (**H**) Capillary sprouting was assessed in patient adipose samples cultured for 2 weeks. (**I**) Total sprouting area at the final measurement (14–15 days after plating) was suppressed in adipose samples obtained from patients with BC mid CTx but returned to baseline values 1 month after CTx. *P < 0.05 versus Pre CTx, mixed model analysis (**A–D**, **G**, and **I**) or 1-tailed unpaired *t* test (**E** and **F**).

Microvascular vasomotor function and angiogenic potential were evaluated in subsets of patients before, during, and 1 month after completion of the final dose of Dox or TZM (Figure 1). Prior to CTx, patients with BC had normal microvascular endothelial function. Endothelium-dependent dilation in response to flow (flow-mediated dilation [FMD]) or acetylcholine (ACh) was severely diminished in vessels obtained during and 1 month after CTx (Figure 1, A–D). After CTx, flow-induced nitric oxide (NO) production was impaired, and mitochondrial hydrogen peroxide (H_2_O_2_) production was elevated (Figure 1, E and F, and Supplemental Figure 1; supplemental material available online with this article; https://doi.org/10.1172/jci.insight.194316DS1). Endothelium-independent vasodilation to the smooth muscle agonist papaverine was normal throughout treatment (Figure 1G). We utilized an established method (22) to evaluate angiogenic potential in adipose samples obtained from patients before, during, and after CTx. Total capillary sprouting area was reduced in adipose samples obtained during CTx and recovered 1 month after CTx (Figure 1, H and I).

### Endothelial dysfunction persists months after treatment cessation.

To supplement findings from the longitudinal study, additional tissue samples were obtained from cross-sectional cohorts of BC survivors 1–24 months after completing CTx and from a group who were CTx-naive at the time of tissue acquisition. Patients were grouped according to the length of recovery time after completion of CTx at the time of sample acquisition (CTx-naive and 1, 2–9, and 12–24 months after CTx). There was no difference in age, racial or ethnic distribution, or cardiovascular risk factors between groups (Supplemental Table 1; all *P* > 0.05).

**Figure 2:**
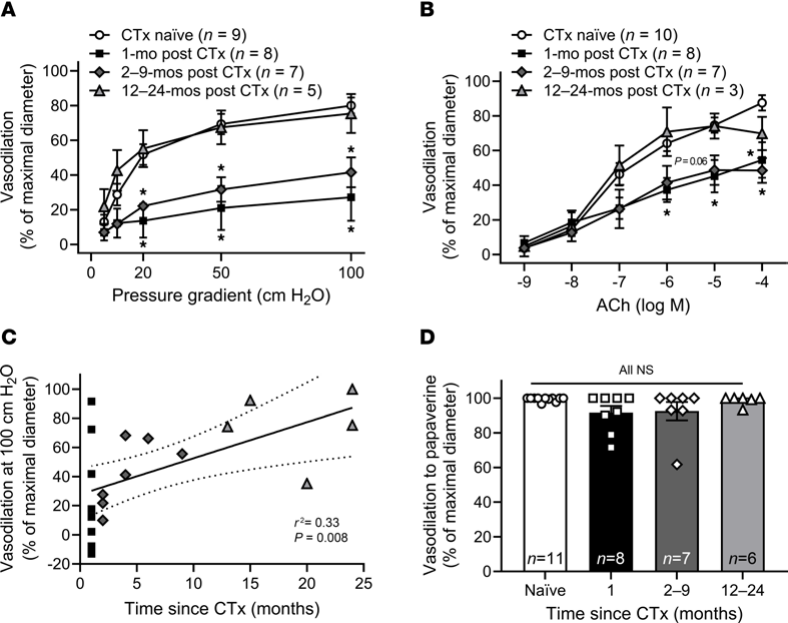
Microvascular endothelial dysfunction persists after treatment cessation. (**A** and **B**) In a cross-sectional study of patients with BC grouped by time since cessation of anticancer therapy (CTx), endothelium-dependent dilation to flow (**A**) and acetylcholine (ACh) (**B**) was impaired to a similar extent in adipose arterioles obtained from patients after 1 month or 2–9 months of recovery after CTx cessation. In vessels obtained from patients 12–24 months after CTx, microvascular endothelial function resembled that of CTx-naive patients with BC (**A** and **B**). (**C**) There was a significant correlation between the time since treatment cessation and maximal flow-mediated dilation at 100 cm H_2_O. (**D**) Endothelium-independent dilation to papaverine did not differ between groups. *P < 0.05 versus CTx naive, 1-way RM-ANOVA or mixed model analysis.

Endothelial function was impaired in arterioles collected 1–9 months after the patient’s last dose of Dox or TZM (Figure 2, A and B). Vasodilation recovered by 12–24 months, and maximal FMD correlated with the length of recovery time post CTx at the time of sample acquisition (Figure 2C). Endothelium-independent vasodilation to papaverine did not differ between groups (Figure 2D).

### Ex vivo exposure to cardiotoxic therapies induces microvascular dysfunction.

We next sought to determine whether ex vivo exposure to clinically relevant doses of cardiotoxic CTx agents (23–25) induces microvascular endothelial dysfunction in healthy tissue. Adipose arterioles isolated from healthy donors (≤1 cardiovascular risk factor; no history of CTx) were incubated with clinically relevant doses of CTx agents with known clinical risk of cardiotoxicity (Dox, TZM). PTX was included as a “negative control” CTx condition due to its minimal clinical risk of cardiotoxicity. Tissue donor characteristics for healthy patients whose vessels were exposed to CTx agents ex vivo are provided in Supplemental Table 2. There was no difference in age, racial or ethnic distribution, or the presence of risk factors in donors whose tissues were used for ex vivo experiments (all *P* > 0.05).

**Figure 3:**
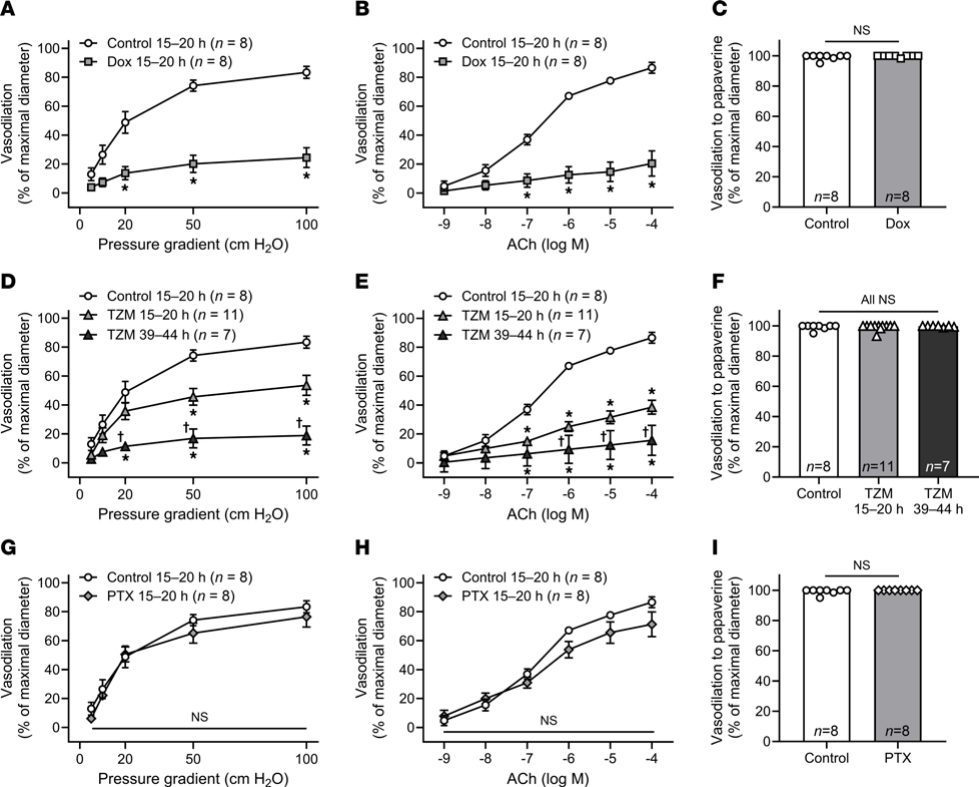
Ex vivo exposure to CTx induces microvascular dysfunction in healthy arterioles. (**A**, **B**, **D**, and **E**) Endothelium-dependent vasodilation to flow and acetylcholine (ACh) was impaired in healthy human adipose arterioles exposed to the cardiotoxic CTx agents doxorubicin (Dox) (**A** and **B**) or trastuzumab (TZM) (**D** and **E**) overnight (15–20 hours). Endothelial function was further suppressed following 2 nights (39–44 hours) of exposure to TZM (**D** and **E**). (**G** and **H**) Vasodilatory responses to flow and ACh were preserved in vessels exposed to paclitaxel (PTX) overnight. (**C**, **F**, and **I**) Endothelium-independent vasodilation to papaverine was not affected by exposure to CTx. The same control data are plotted for each drug. *P < 0.05 versus Control, ^†^*P* < 0.05 versus TZM 15–20 hours, 2-way repeated-measures ANOVA (**A**, **B**, **D**, **E**, **G**, **H**), 1-way ANOVA (**F**), 2-tailed t test (**I**), or mixed model analysis.

Overnight exposure (15–20 hours) of healthy arterioles to Dox nearly abolished endothelium-dependent vasodilation (Figure 3, A and B, and Supplemental Figure 2, A and B) without affecting smooth muscle vasodilation to the endothelium-independent dilator agent papaverine (Figure 3C). Overnight exposure to TZM also induced endothelial dysfunction, which was exacerbated with longer exposure duration (2 nights; 39–44 hours) (Figure 3, D and E, and Supplemental Figure 2, C and D). We previously confirmed in pilot studies that microvascular endothelial function is preserved in control vessels incubated in media for 2 nights (26). TZM did not affect smooth muscle dilation to papaverine (Figure 3F). In contrast to Dox and TZM, exposure to PTX, which poses minimal clinical risk of cardiotoxicity, did not affect endothelium-dependent vasodilation (Figure 3, G and H, and Supplemental Figure 2, E and F) or smooth muscle dilation to papaverine (Figure 3I).

**Figure 4:**
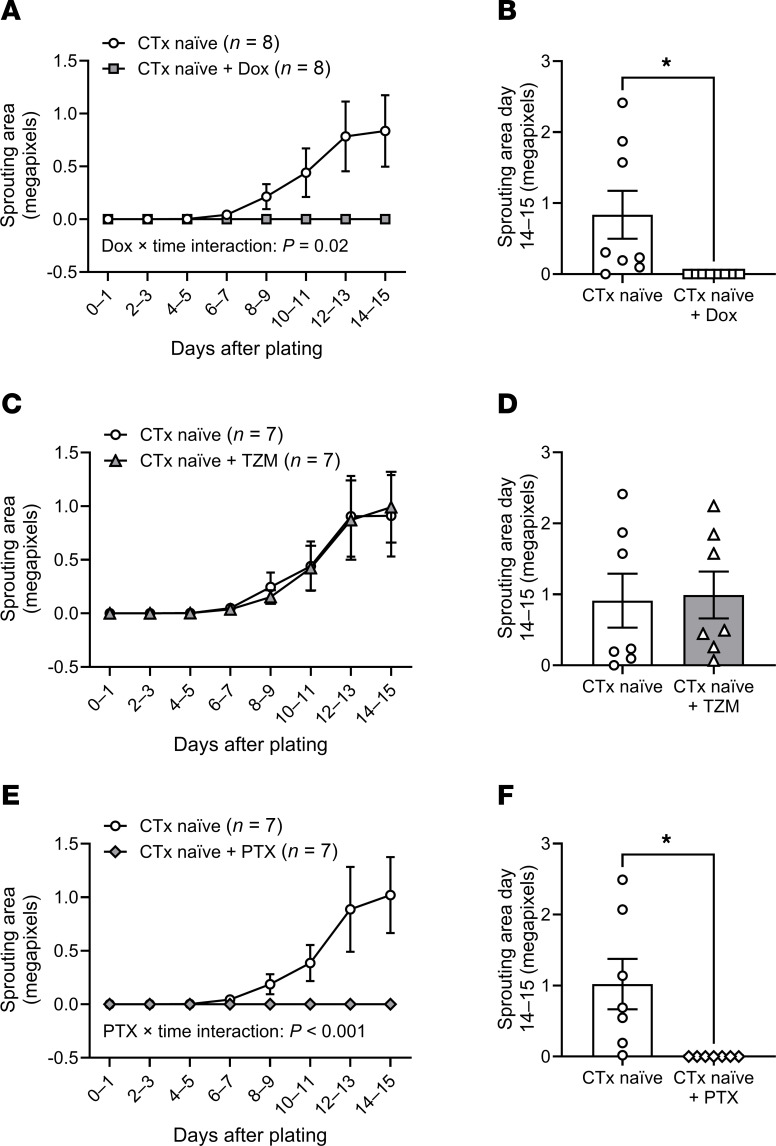
Angiogenic potential after ex vivo exposure to CTx. Capillary sprouting was assessed in paired adipose samples from CTx-naive patients with BC under control conditions and during ex vivo exposure to doxorubicin (Dox, 100 nM), trastuzumab (TZM, 10 μg/mL), or paclitaxel (PTX, 1 μM). (**A**, **B**, **E**, and **F**) Dox and PTX suppressed the total capillary sprouting area over time (**A** and **E**) and sprouting area at the final measurement (14–15 days after plating) (**B** and **F**). (**C** and **D**) TZM did not affect capillary sprouting area. Significant interaction effects in r mixed model analyses are reported in **A**, **C**, and **E**. Two-tailed *t* test was used in **A**, **C**, and **E**. *P < 0.05, paired *t* test (**B**, **D**, and **F**).

To determine whether ex vivo exposure to CTx agents disrupts angiogenic potential, we incubated adipose samples from CTx-naive patients with BC with Dox, TZM, or PTX. Incubation with Dox or PTX, agents known to induce cell cycle arrest (27–29), abolished capillary sprouting (Figure 4, A, B, E, and F), whereas incubation with the targeted molecular therapy TZM did not affect angiogenic potential (Figure 4, C and D).

### Differential gene expression in ECs exposed to CTx.

**Figure 5:**
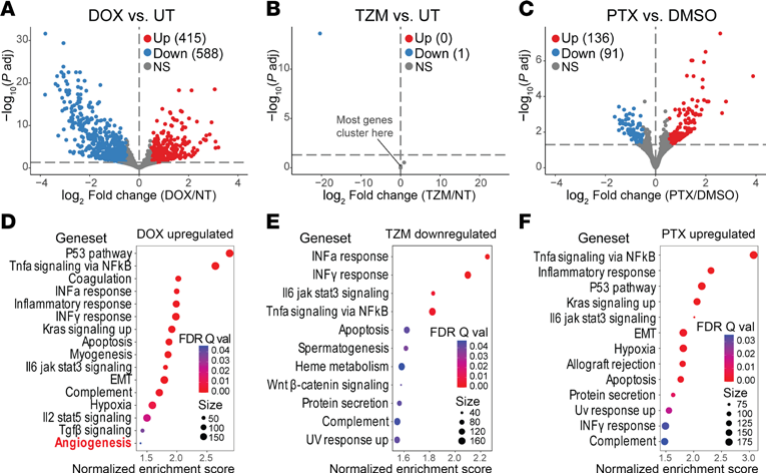
CTx alters endothelial cell gene expression. (**A**–**C**) Volcano plots show differentially expressed genes as determined by DESeq2 (*P*_adj_ < 0.05 and abs[log_2_ fold change] > 0.5) in endothelial cells derived from 5 donors without known cardiovascular disease following ex vivo exposure to CTx agents. (**D**–**F**) Dot plots show gene sets that were significantly enriched and upregulated in doxorubicin-treated (**D**, Dox, *n* = 5) and paclitaxel-treated (**F**, PTX, *n* = 5) cells, or those significantly enriched and downregulated in trastuzumab-treated cells (**E**, TZM, *n* = 4) by GSEA analysis (FDR *q* < 0.05). No gene sets were found upregulated in TZM.

To investigate the effect of CTx agents on EC gene expression, bulk RNA-Seq was performed on cultured, patient-derived ECs from 5 donors (Supplemental Table 3). Microvascular ECs were isolated from adipose tissue, and EC identity was confirmed via FACS prior to exposure to Dox, TZM, PTX, or corresponding vehicle controls. Differential gene expression analysis with DESeq2 (Figure 5, A–C, *P*_adj_ < 0.05 and abs[log_2_ fold change] > 0.5) (30) identified the most differentially expressed genes (DEGs) with Dox treatment (415 upregulated [up], 588 downregulated [down]; Figure 5A) followed by PTX (136 up, 91 down; Figure 5C). Treatment with the targeted molecular therapy TZM resulted in the fewest transcriptome changes compared with control (0 up, 1 down; Figure 5B). Differential gene expression analysis with EdgeR (*P*_adj_ < 0.05 and abs[log_2_ fold change] > 0.5) (31) confirmed the results by showing 1 DEG with TZM treatment and > 84% overlapping DEGs from DESeq2 with Dox and PTX treatments (Supplemental Table 4). To understand pathways affected by CTx treatments, we performed gene set enrichment analysis (GSEA) to detect coordinated expression changes in defined gene sets (Figure 5, D–F, and Supplemental Figure 3, A and B; gene sets with FDR *q* < 0.05 considered significant) (32, 33). Among gene sets significantly enriched and upregulated in Dox-treated cells, we found “angiogenesis” (Figure 5D and Supplemental Figure 3C). Further investigation revealed elevated mRNA expression of VEGFR-2 (gene name KDR) and NRP1 (coreceptor of VEGFRs) with a possible but nonsignificant trend toward increased expression of VEGFR-1 (gene name *Flt1*; *P* = 0.096) (Supplemental Figure 3, D–G). GSEA also indicated enrichment of inflammatory gene sets such as “TNF-α signaling via NF-κB” and “inflammatory response” in Dox- and PTX-treated ECs (Figure 5, D and F), whereas “TNF-α signaling via NF-κB” was suppressed in TZM-treated cells compared with vehicle (Figure 5E).

### Tissue gene expression in patients with BC exposed to in vivo therapy.

**Figure 6:**
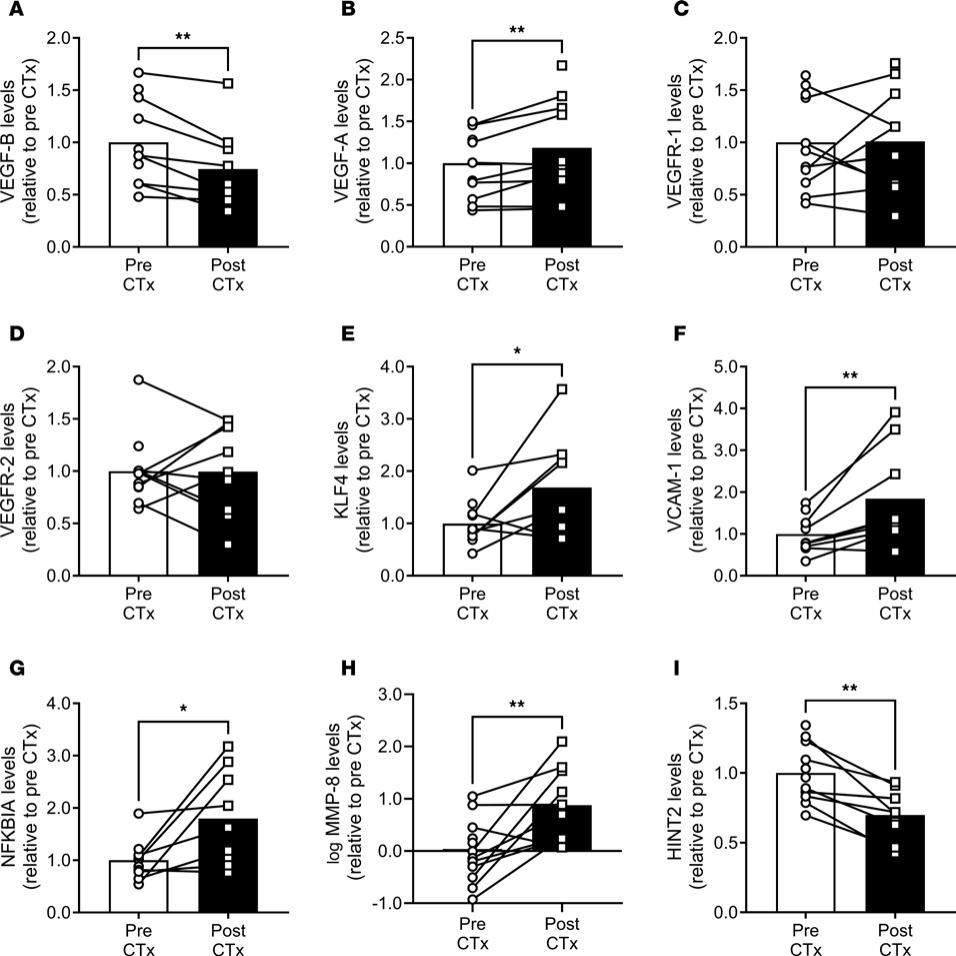
Effect of CTx on adipose gene expression in patients with BC. (**A**–**I**) Expression of genes related to vascular endothelial growth factor (VEGF) signaling, angiogenesis, endothelial function, and inflammation is shown for adipose samples obtained in a longitudinal study of patients with BC before and 1 month after CTx treatment. Ct values were normalized to 18s, before being normalized to the group average pre-CTx value using the 2^−ΔΔCt^ method. MMP-8 expression values were log-transformed prior to analysis. *P < 0.05, **P < 0.01, 1-tailed paired *t* test.

We next evaluated molecular changes related to VEGF signaling, angiogenesis, and inflammation in adipose samples obtained from longitudinal study participants before and after clinical CTx regimens. VEGF-B gene expression was reduced post CTx (Figure 6A), while VEGF-A expression was upregulated (Figure 6B). Expression of VEGFR-1 and VEGFR-2 did not change in adipose samples after treatment (Figure 6, C and D). Krüppel-like factor 4 (KLF4), a transcription factor that regulates VEGF, angiogenesis, and vascular inflammation (34, 35), increased after CTx (Figure 6E). Expression of the inflammatory mediator vascular cell adhesion molecule 1 (VCAM-1) was increased post CTx (Figure 6F), as was NF-κB inhibitor α (NFKBIA) (Figure 6G), which sequesters NF-κB in an inactive state. We also observed a robust elevation in matrix metalloproteinase 8 (MMP-8) (Figure 6H), a collagenase involved in EC sprouting and proliferation (36), after CTx. Expression of histidine triad nucleotide binding protein 2 (HINT2), a mitochondrial protein with EC-protective effects (37), decreased after CTx (Figure 6I).

Gene expression in microvessels obtained from longitudinal study participants is summarized in Supplemental Figure 4. There was a trend toward reduced VEGF-B expression in microvessels after CTx that did not reach statistical significance (Supplemental Figure 4A, *P* = 0.06). Expression of VEGF-A and VEGFR-1 were unchanged (Supplemental Figure 4, B and C), and there was a nonsignificant trend toward increased expression of VEGFR-2 in microvessels after CTx (Supplemental Figure 4D, *P* = 0.06). There was a small, significant increase in expression of major histocompatibility complex (MHC) class II DM α chain (HLA-DMA1) (Supplemental Figure 4E), which enables protein loading of MHC II molecules to facilitate antigen presentation and induce local inflammation. There was also a nonsignificant trend toward reduced expression of HINT2 in microvessels (Supplemental Figure 4F; *P* = 0.07), which mirrors the observation in adipose tissue (Figure 6I).

### VEGF-B treatment prevents Dox- and TZM-induced microvascular toxicity ex vivo.

**Figure 7:**
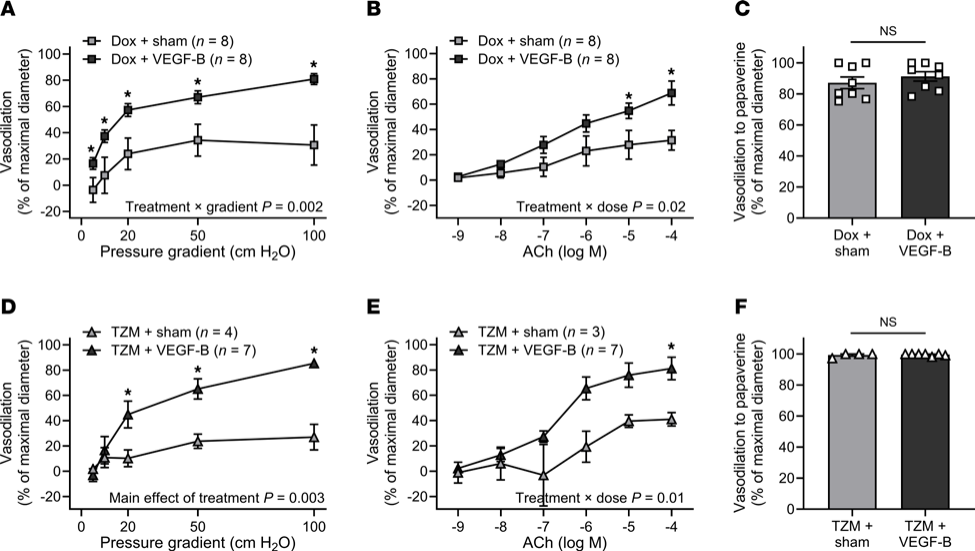
VEGF-B treatment prevents doxorubicin- and trastuzumab-induced microvascular endothelial dysfunction ex vivo. (**A** and **B**) In paired adipose arterioles from healthy human donors, treatment with conditioned media containing VEGF-B mitigated the effect of overnight (15–20 hours) exposure to doxorubicin (Dox, 100 nM) on flow-mediated vasodilation (FMD) (**A**) and acetylcholine-induced (ACh-induced) vasodilation (**B**) compared with sham conditioned media. The 1 × 10^−4^ M ACh dose was not evaluated in all samples. (**C**) Endothelium-independent dilation to papaverine did not differ between treatments. (**D**–**F**) VEGF-B treatment also mitigated the effect of 2 nights (39–44 hours) of exposure to trastuzumab (TZM, 10 μg/mL) on FMD (**D**) and ACh-mediated dilation (**E**) without impacting dilation to papaverine (**F**). *P < 0.05 versus sham, 2-way repeated-measures ANOVA, mixed model analysis, or 2-tailed paired *t* test.

Prior evidence that VEGF-B protects against Dox-induced cardiotoxicity in mice (13), together with results from GSEA analysis and reduced VEGF-B expression in patients with BC after CTx (Figure 6A), led us to explore the utility of VEGF-B before treatment to prevent the detrimental effects of Dox or TZM exposure on human microvascular function ex vivo. We did not investigate the effects of VEGF-B before treatment on vessels exposed to PTX because ex vivo exposure to PTX did not induce microvascular dysfunction (Figure 3, G–I). Isolated arterioles from healthy donors were incubated with conditioned media containing VEGF-B protein or corresponding sham-conditioned media (Supplemental Figure 5) prior to incubation with CTx agents. Incubation with sham conditioned media did not alter the effects of Dox or TZM on endothelial function (Supplemental Figure 6). In arterioles from healthy donors, treatment with conditioned media containing VEGF-B protein fully prevented the effect of exposure to Dox (Figure 7, A and B) or TZM (Figure 7, D and E) on endothelial function without affecting smooth muscle dilator capacity (Figure 7, C and F).

## Discussion

To our knowledge, the present study provides the first comprehensive, longitudinal investigation of microvascular endothelial function in patients with BC undergoing clinical treatment with anthracyclines and/or anti-HER2 therapy. We describe the direct effect of 3 CTx agents commonly used to treat BC on vasomotor function, angiogenesis, gene expression, and RNA-Seq. The results demonstrate detrimental effects of cardiotoxic antineoplastic therapies on the human microvascular endothelium when administered in vivo or ex vivo, which may contribute to treatment-induced cardiovascular toxicity. For the first time to our knowledge, we directly demonstrate a longitudinal decline in microvascular endothelial function in patients with BC treated with Dox and/or anti-HER2 therapy, which is associated with suppressed NO production, elevated H_2_O_2_ levels, and impaired angiogenic potential. The findings of our cross-sectional study of patients with BC indicate that microvascular dysfunction persists for at least 9 months after CTx cessation. RNA-Seq in patient-derived ECs exposed to CTx agents identified enrichment of gene sets including angiogenesis and inflammatory signaling in cells exposed to Dox, which highlights molecular targets that warrant further exploration. These findings are supported by a longitudinal decline in adipose gene expression of VEGF-B coupled with increased adipose expression of inflammatory markers in patients with BC following treatment with Dox and/or anti-HER2 therapy. Ex vivo VEGF-B treatment prevents the direct effect of Dox or TZM on vasomotor function in arterioles, which provides rationale for future investigations of targeted therapy as a means to prevent CTx-induced vascular toxicity. Collectively, these findings implicate CTx-induced endothelial dysfunction as a consequence of cardiotoxic antineoplastic therapies and highlight the microcirculation as a potential strategic target to prevent or predict adverse cardiovascular outcomes in patients with BC.

### Endothelial dysfunction induced by CTx.

In our longitudinal study of patients with BC, we observed severe microvascular endothelial dysfunction during CTx that did not improve 1 month after treatment cessation, and cross-sectional analyses suggest that impairments persist at a similar magnitude for ≥ 9 months. Whereas vasomotor impairments persisted months after treatment cessation, angiogenesis was only suppressed during treatment. These data suggest that angiogenic potential recovers sooner than endothelium-dependent vasomotor function. In several pathological states associated with microvascular dysfunction, the maximal change in diameter to a flow stimulus is maintained, yet the underlying mediator of FMD undergoes a pathological switch from NO to H_2_O_2_ (38, 39). Thus, although the cross-sectional study suggests endothelium-dependent dilation largely recovers by 12–24 months after treatment, future studies are warranted to investigate whether restored dilation reflects a healthy, NO-mediated phenotype versus an H_2_O_2_-mediated phenotype similar to that observed in patients with coronary artery disease (CAD) (38). Future studies are also needed to determine whether microvascular toxicity directly contributes to CTx-induced cardiac dysfunction.

To our knowledge, this is the first study to demonstrate a direct effect of CTx agents on the human microcirculation. Ex vivo exposure to CTx agents recapitulated clinical exposure, which highlights this model as a tool to investigate underlying mechanisms and potential therapeutic interventions in future studies. A crucial limitation of patient research is that detailed mechanistic studies are not always feasible in patients: for instance, it is not possible to isolate the effects of a single drug in a patient whose clinical therapy requires treatment with multiple types of CTx. The isolated vessel model allows direct measures of microvascular function in patients undergoing clinical treatment, and data from the ex vivo exposure studies indicate that this may be a valuable model to investigate mechanisms in human arterioles.

### Effects on vasomotor function versus angiogenesis.

Specific CTx agents have distinct effects on vasomotor function versus angiogenesis, as Dox suppressed both processes, TZM reduced vasodilatory function without affecting angiogenesis, and PTX suppressed angiogenesis without affecting vasodilatory function. Vasomotor function was suppressed by exposure to the cardiotoxic agents Dox and TZM but was preserved with PTX, which was included as a negative control CTx agent due to its minimal clinical risk of cardiotoxicity. The effects of CTx agents on angiogenesis did not align with known risk of cardiotoxicity associated with each CTx agent. This raises the possibility that detrimental effects of CTx agents on microvascular vasomotor function may be more important in promoting cardiotoxicity compared with suppression of angiogenesis, though further research is required to investigate the relative contribution of vasomotor function versus angiogenesis to cardiotoxicity.

The mechanisms underlying the divergent effects of CTx agents on vasomotor function versus angiogenesis are unclear and likely related to the cellular effects of each agent. Dox causes severe cellular damage, including DNA damage and oxidative stress, which prevents cell division and suppresses formation of new blood vessels. PTX interferes with mitosis via microtubule stabilization, thus inhibiting cell division and angiogenesis. We hypothesize that vasomotor function is preserved in vessels exposed to PTX because ECs within established arterioles are nondividing. TZM is a monoclonal antibody that specifically targets HER2, which is not directly involved in cell division, and we did not observe an effect of TZM on capillary sprouting in the present study. Dox and TZM both cause mitochondrial dysfunction (40–42), and mitochondrial H_2_O_2_ production was greater in arterioles isolated from patients with BC after treatment with anthracyclines and/or anti-HER2 therapy in our longitudinal study. Recent evidence indicates that mitochondrial ATP production is critical to maintaining vasodilator capacity in rodent models (43). Further studies are needed to determine whether the mitochondrial effects of Dox and TZM contribute to functional impairments in endothelium-dependent vasodilation.

### Clinical relevance of microvascular dysfunction.

As the primary site of vascular resistance, arterioles play a key role in regulating tissue perfusion; thus, microvascular health is crucial for overall cardiovascular health. Endothelial dysfunction is an independent predictor that contributes to cardiovascular risk, and impaired peripheral vasoreactivity may contribute to hypertension, lymphedema, and/or reductions in perfusion of vital organs that commonly occur in patients with BC. In both the general population and in patients with BC, coronary microvascular dysfunction is among the best predictors of major adverse cardiovascular events (8, 44). A recent prospective study of patients with BC reported a reduction in coronary fractional flow reserve (CT-FFR) of epicardial arteries after anthracycline therapy using noninvasive computed tomography angiography (45). The authors identified low baseline CT-FFR of the left anterior descending artery as the best predictor of developing CTx-related cardiac dysfunction among parameters tested (45), which highlights the clinical relevance of the coronary vasculature to CTx-induced cardiac dysfunction. The relationship between CT-FFR and endothelial function is not well defined, and the extent to which CT-FFR results reflect impairments in large artery versus microvascular function is unclear. Importantly, Dox and TZM both have direct effects on cardiomyocytes, and the vascular toxicity observed in the present study represents only 1 of many factors that likely contribute to CTx-induced cardiotoxicity.

The results of the present investigation indicate that Dox induces endothelial toxicity in the adipose microcirculation. The magnitude of vasomotor impairment in adipose arterioles exposed to Dox ex vivo in the present study is consistent with our previous results in human coronary arterioles (11), which suggests that CTx induces systemic microvascular dysfunction and highlights the potential utility of adipose arterioles as a surrogate for coronary microvascular function for monitoring purposes or as a model for future mechanistic studies. While the relationship between adipose microvascular function, coronary microvascular function, and cardiac contractile function requires future study, data from the past decade suggest that the peripheral microcirculation is an adequate and easily accessible surrogate for the coronary circulation (46). Whether the coronary microcirculation is directly affected by clinical exposure to CTx in humans has not been established. However, changes in the peripheral microcirculation could indirectly contribute to cardiac dysfunction via reductions in perfusion and/or increased systemic vascular resistance.

Our observation that Dox impairs microvascular function in patients with BC is in line with reports of microcirculatory damage in animal models of Dox-induced cardiotoxicity (12, 47). A recent study demonstrated degradation of reactive hyperemia indices measured via peripheral arterial tonometry in patients with BC undergoing combined anti-HER2 and anthracycline treatment (48). Moreover, the authors reported a significant correlation between peripheral microvascular reactivity and changes in left ventricular ejection fraction, which supports the notion that systemic microcirculatory dysfunction may contribute to CTx-induced cardiotoxicity (48). Although we observed a direct effect of TZM on microvascular endothelial function in the present study, Hazim et al. reported no change in peripheral microvascular reactivity in patients treated with anti-HER2 therapy who did not receive anthracyclines (48). Peripheral arterial tonometry is an indirect measure of microvascular function that differs physiologically from conventional assessments of endothelial function (48–50); thus, our observations using sensitive, direct measures of microvascular endothelial function add to these findings and reveal TZM-induced impairments in endothelium-dependent vasodilation.

A recent cross-sectional study by Szczepaniak et al. reported impaired endothelium-dependent dilation in arteries obtained from patients with BC 1 month after chemotherapy compared with chemotherapy-naive patients (15). The authors reported greater production of reactive oxygen species and a lower contribution of NO synthase to endothelium-dependent dilation, which aligns with our observations of augmented H_2_O_2_ production and suppressed NO formation in the microvasculature of patients with BC following CTx. In the present study, ex vivo exposure of arterioles (~170 μm) to 100 nM Dox had a profound effect on endothelium-dependent dilation, whereas in the Szczepaniak paper, ex vivo exposure of larger arteries (~1,000 μm) to 100 nM Dox did not yield statistically significant changes in dilation to ACh (*n* = 5) (15). Although the reasons for this discrepancy are unclear, it is possible that arterioles are more susceptible to acute Dox toxicity than upstream conduit arteries. Other studies have reported impaired conduit artery endothelial function in patients and animal models exposed to Dox (40, 51–53), so future studies that directly compare the effects of Dox on the microcirculation versus conduit arteries are warranted.

Taxanes pose minimal risk of serious cardiotoxicity (6, 54), and we did not observe vasomotor dysfunction in adipose arterioles from our patient population exposed to PTX. Interestingly, Szczepaniak et al. identified docetaxel-induced vascular toxicity in arteries from humans and rodents (15). Several factors may contribute to the divergent effects of taxanes on vasomotor function between studies, such as the specific drug investigated (docetaxel versus PTX, which differ in cytotoxicity; ref. 55) and location along the vascular tree. Whether longer exposure to PTX might affect microvascular endothelial function remains unclear; however, PTX is not associated with a high risk of clinical cardiotoxicity.

### Time course of microvascular dysfunction.

In patients with BC, Fredslund et al. (56) observed no difference in the forearm blood flow response to intraarterial infusion of ACh 2 weeks after treatment with epirubicin, cyclophosphamide, and docetaxel or PTX, but the response was diminished 1 year after CTx, which was accompanied by a lesser contribution of NO synthase to dilation. Although direct comparison with present study is not possible due to differences in the timeline, treatment regimen, and experimental protocol, it is notable that vascular function was preserved 2 weeks after treatment and subsequently declined in the study by Fredslund et al. (56). We observed severe endothelial dysfunction during and 4 weeks after CTx, which may be linked to the greater risk of cardiotoxicity with Dox compared with epirubicin.

### Molecular changes.

This is the first reported comprehensive analysis to our knowledge of DEGs and GSEA resulting from treatment of patient-derived microvascular ECs treated with various CTx agents. RNA-Seq results demonstrate a direct effect of CTx agents on EC gene expression, and GSEA identified changes fundamental to reported cellular effects of each agent (27–29, 57–59). Dox and PTX are both classic chemotherapies that initiate cell cycle arrest and promote apoptosis (27–29), and the similar mechanisms of Dox and PTX with regard to the cell cycle may underlie the overlap in enrichment of some gene sets and effects on angiogenesis in the present study. In contrast to Dox and PTX, TZM is a monoclonal antibody that specifically targets HER2 signaling with minimal effects on gene expression.

### VEGF signaling.

Our findings that VEGF-B treatment protects human arterioles from exposure to Dox or TZM add to existing evidence that VEGF-B confers endothelium-protective effects capable of mitigating Dox-induced vascular toxicity in rodent and cell culture models (13). VEGF-B, which is highly expressed in the heart, is poised to regulate crosstalk between the coronary microcirculation and cardiomyocytes, and VEGF-B gene therapy protects cardiomyocytes against ischemia-reperfusion injury and upregulates eNOS expression (60, 61). Adipose VEGF-B expression was reduced in patients with BC after CTx. Importantly, in rodents, VEGF-B overexpression does not interfere with the efficacy of Dox to combat tumor growth (13, 62), while VEGF-B deficiency accelerates tumor growth (62). Despite suppressing tumor growth, tumor VEGF-B overexpression may promote metastasis (63); thus, strategies that harness VEGF-B must be approached carefully in the context of cancer therapy. The present study was intended to provide proof-of-principle data regarding the feasibility of a targeted intervention to prevent CTx-induced endothelial dysfunction ex vivo, and it is crucial to note that the effect of VEGF-B manipulation on in vivo microvascular function, tumor growth, or CTx efficacy is currently unknown in humans. Evidence for cardiovascular protection conferred by VEGF-B in the current study and prior studies by others warrants further investigation.

Given the divergent effects of Dox and TZM on EC gene expression, it is notable that VEGF-B treatment prevented microvascular dysfunction in vessels exposed to either Dox or TZM. While Dox directly affects gene sets related to VEGF signaling, TZM has minimal effect on EC gene expression, which may reflect the targeted molecular nature of TZM as a monoclonal antibody against HER2 (ErbB2). It is possible that VEGF-B treatment confers nonspecific endothelial protection that is effective in preventing TZM-induced endothelial dysfunction. Another possibility is that VEGF-B treatment interacts with posttranscriptional effects of TZM downstream of diminished ErbB2 signaling. Further research is required to investigate the signaling mechanisms by which VEGF-B treatment protects against CTx-induced vascular toxicity.

VEGF-A signaling has been implicated in Dox-induced cardiotoxicity in preclinical models (13, 47, 64–66). Dox has been shown to suppress VEGF-A expression (47, 66), increase VEGF-A release from ECs (65), and reduce expression of VEGFR2 in the heart (65). In mice, VEGF-A overexpression protects against Dox-induced vascular injury and cardiotoxicity, whereas VEGF-A siRNA exacerbates Dox-induced endothelial toxicity (47, 66). We observed increased VEGF-A expression in adipose samples from patients with BC 1 month after CTx, and there was a trend toward increased microvascular VEGFR2 expression.

### Inflammatory mediators.

We observed increased expression of several genes related to inflammation and the immune system in patients with BC following therapy, including VCAM-1, NFKBIA, HLA-DMA1, and MMP-8. MMP-8 is a pleiotropic collagenase involved in angiogenesis that has been implicated in atherogenesis (67) and tumor suppression (68). Plasma MMP-8 levels are increased in patients with atherosclerotic disease (69, 70), and serum MMP-8 concentration is an independent predictor for myocardial infarction and CAD (71). Given the robust elevation in adipose MMP-8 expression in the present study, further investigation is warranted regarding the potential value of plasma MMP-8 as a biomarker for CTx-induced cardiovascular toxicity.

Expression of HINT2, an inner mitochondrial membrane protein that modulates mitochondrial dynamics and Ca^2+^ handling, declined in patients with BC during and after CTx. In mice, EC-specific overexpression of HINT2 protects coronary microvascular function and preserves angiogenic potential with ischemia-reperfusion injury (37). HINT2 enhances VEGFR2 expression in coronary microvascular ECs and suppresses VCAM-1 expression (37), which aligns with findings of the present study wherein HINT2 expression was reduced while VCAM-1 expression was increased in patients following CTx.

### Study limitations.

Clinical treatment regimens varied between patients, and we cannot exclude that other CTx agents, lifestyle, environmental, or socioeconomic factors contributed to the findings of our longitudinal and cross-sectional studies. Genetic polymorphisms are associated with the risk of radiation damage to the vasculature (72), and genetic predisposition and social determinants of health contribute to MACE in cancer survivors (73, 74). However, the present study was not powered to investigate potential genetic factors that may influence the risk of CTx-induced microvascular dysfunction. Our study primarily focused on female patients and tissue donors to reflect the demographics of patients affected by BC, who are predominantly female. We included patients with preexisting conditions and comorbidities, such as tobacco use, hypertension, and diabetes, to better reflect the typical patient with BC. Future studies are warranted to determine whether similar effects are observed in other cancer types, with different CTx agents, and/or in male patients. Further research is also needed to investigate how CTx-induced vascular toxicity interacts with preexisting cardiovascular morbidity and whether endothelial dysfunction is associated with cardiovascular events.

Several aspects of the present study are limited by small sample size. Mid-CTx visits were halted due to COVID-19, which limits our ability to detect statistical differences and precludes subgroup analyses to investigate differences between treatment regimens (e.g., patients treated with Dox versus TZM versus Dox + TZM). Future studies with larger cohorts are warranted to compare the longitudinal effect of different treatment regimens on microvascular function. Due to limitations in sample size for the longitudinal study, we were unable to evaluate gross morphological changes that may contribute to the observed microvascular dysfunction via histological measures. Given the established adverse effects of some CTx agents on vascular endothelial barrier function and promotion of perivascular fibrosis, such studies are warranted to further understand the underlying pathology.

The isolated arteriole model enables controlled, mechanistic investigations that are not feasible in human participants; however, it does not fully replicate in vivo hemodynamics or systemic factors that may influence microvascular function. Although ex vivo exposure to CTx agents recapitulated clinical exposure in the present study, our findings are limited by the acute time course of ex vivo exposure. Ex vivo studies also exclude systemic factors, which may influence the effect of CTx on endothelial function and angiogenesis, such as circulating molecules, tumor cells, or the tumor microenvironment, which warrant future investigation. Moreover, in part due to the limited follow-up of patients in longitudinal study, the present study did not investigate whether declines in adipose microvascular function are associated with subsequent development of cardiac contractile dysfunction or major adverse cardiovascular events, which usually manifest years after cessation of therapy (75). Finally, our results related to VEGF-B treatment are limited to Dox- and TZM-induced vasomotor dysfunction, as we did not evaluate angiogenesis or PTX with VEGF-B treatment.

### Conclusions.

The present study demonstrates that cardiotoxic antineoplastic agents provoke endothelial dysfunction in the human microcirculation. Vasomotor function is severely impaired in patients with BC during neoadjuvant therapy, and endothelial dysfunction persists for at least 9 months after treatment cessation. The findings highlight the microcirculation as a potential target for therapeutic intervention to reduce the cardiovascular complications associated with CTx.

## Methods

### Sex as a biological variable

The present study focused on BC, which predominantly affects female patients; thus, all participants in the longitudinal and cross-sectional protocols were female. In accordance with this, the vast majority of ex vivo incubation studies were also performed in arterioles from female tissue donors (Supplemental Table 2).

### Study design

The objectives of this study were to define the effect of clinically used CTx on the human microcirculation, identify related molecular changes, and determine whether acute vascular toxicity can be prevented. Patients with BC were enrolled in a longitudinal study of microvascular function (Protocol I) utilizing isolated microvessels, angiogenesis assays, and gene expression from adipose biopsies before, during, and after in vivo CTx. To complement the longitudinal study, we investigated microvascular function in otherwise-discarded surgical samples to evaluate the chronic effect of clinical exposure to CTx utilizing a cross-sectional study of patients with BC (Protocol II) and determined the effect of ex vivo exposure to CTx on healthy microvessels (Protocol III). Patient-derived ECs were exposed to CTx ex vivo for RNA-Seq to investigate molecular changes. Additional experimental details are provided for each protocol below as well as in Supplemental Methods.

#### Protocol I, a longitudinal study.

Women diagnosed with BC were enrolled in a longitudinal study to evaluate the effects of in vivo clinical exposure to CTx. All participants received standard-of-care therapy including Dox and/or HER2 monoclonal antibody therapy (e.g., TZM). Further details regarding inclusion and exclusion criteria are provided in the Supplemental Methods. Adipose biopsies were obtained from the site of port insertion/removal or via a gluteal biopsy at 3 time points: (a) baseline, immediately prior to initiation of CTx, (b) during CTx, and (c) approximately one month after treatment cessation (i.e., the final dose of Dox or TZM). Samples were promptly suspended in HEPES buffer, stored on ice, and transported to the laboratory for dissection and experiments to assess vasomotor function, angiogenic potential, and gene expression.

#### Protocol II, a cross-sectional study.

Deidentified adipose samples were obtained from separate cohorts of BC survivors undergoing surgical procedures (e.g., breast reconstruction) at time points 1–24 months after completing CTx and a group who were CTx-naive at the time of surgery. Samples were promptly suspended in HEPES buffer, stored on ice, and transported to the laboratory to for dissection and experiments to assess vasomotor function. Cross-sectional analyses were performed by grouping data from patients according to the length of recovery time post CTx (4 groups: CTx-naive and 1, 2–9, and 12–24 months post CTx) at the time of sample acquisition.

#### Protocol III and ex vivo exposure.

Deidentified surgical discard tissue from healthy patients (≤1 cardiovascular risk factor; no history of CTx) was utilized to investigate direct effects of ex vivo exposure to clinically relevant doses of CTx (23–25) on endothelial function. Adipose arterioles were isolated and incubated with Dox (100 nM), TZM (10 μg/mL), or PTX (1 μM) overnight (15–20 hours) or for 2 nights (39–44 hours) prior to assessing vasomotor function. The 5-hour window, which is used routinely for this model, allows for factors such as variability in the timing of tissue arrival in the laboratory and time required to set up isolated vessel preparations. We have previously established viability of isolated vessels for at least 48 hours after sample collection (26, 76).

### Vasomotor function

Arterioles were dissected and cannulated for videomicroscopy as previously published (26). Briefly, adipose arterioles (50–250 μm inner diameter) were carefully dissected, cleaned of connective tissue and adipose, cannulated on glass micropipettes within a tissue chamber, and pressurized to 60 mmHg. After equilibration and pre-constriction with endothelin-1, vasomotor function was evaluated in response to endothelium-dependent and -independent stimuli. In separate experiments, endothelium-dependent vasodilation was assessed in response to flow (FMD) or ACh, and endothelium-independent vasodilation was assessed in response to the vascular smooth muscle agonist papaverine. Fluorescent probes were utilized to quantify NO and mitochondrial H_2_O_2_. Each measure of vasomotor function reflects 1 replicate per patient. To maintain scientific rigor and reproducibility, vessels were not included in analyses if any of the following parameters were not met: maximum inner diameter ≤ 250 μm, pre-constriction with endothelin-1 ≤ 20% or >75% (based on maximal diameter), and matched impedance of cannulae verified by ≤ 5 μm difference in inner diameter at maximal flow (100 cm H_2_O pressure gradient) in either direction. Further experimental details are provided in the Supplemental Methods.

### Angiogenesis

Adipose samples (~1 mm^2^ in size) were embedded in Matrigel in a 96-well plate and incubated for 2 weeks to assess angiogenic potential (22). After incubation, 100 μL of media ± CTx agents (500 nM Dox, 10 μg/mL TZM, or 1 μM PTX) were added to wells and changed twice weekly with 8 replicates per patient per condition. Images were taken with a 2× objective every other day, and capillary sprouting area was quantified using ImageJ (NIH) (22). Additional details are provided in the Supplemental Methods.

### RNA-Seq

Microvascular ECs were cultured by Akiko Mammoto’s lab (Medical College of Wisconsin, Milwaukee, Wisconsin, USA), as previously published (77). Patient-derived ECs were isolated from fresh human adipose with magnetic beads containing CD31 antibodies to enrich for ECs. After isolation, endothelial identity was verified by staining for von Willebrand factor followed by FACS analysis with VE-cadherin before culture. Cells were cultured for 3–4 passages to provide sufficient material for bulk sequencing and avoid loss of endothelial identity. ECs were exposed to Dox, TZM, PTX, dimethyl sulfoxide (DMSO, vehicle control for PTX), or media (vehicle control for Dox and TZM) for 24 hours, and total RNA was isolated and sequenced. DEGs were identified in comparison to concurrent vehicle control-treated cells with DESeq2 (30) and EdgeR (31). GSEA was performed to identify coordinated changes in gene sets and pathways (32, 33). More details about primary EC preparation and RNA-Seq can be found in Supplemental Methods.

### Quantitative PCR

mRNA expression levels were quantified via standard methods. Expression levels were normalized to 18S rRNA then normalized to the group average pre-CTx gene expression using the 2^−ΔΔCt^ method.

### Treatment with conditioned media containing VEGF-B

Vessels from healthy donors (≤1 cardiovascular risk factor; no history of CTx; Supplemental Table 2) were treated with conditioned media containing VEGF-B protein. Recombinant VEGF-B protein and a corresponding sham were generated using 293T cells transduced with an adeno-associated viral vector expressing VEGF-B_186_ or a control vector containing scrambled sequence (13). The 293T cells were washed to remove viral vectors, before conditioned media containing VEGF-B or sham were collected. VEGF-B protein expression was confirmed in the conditioned media via Western blot (Supplemental Figure 5). Vessels were incubated in 1 mL of VEGF-B–containing or sham conditioned media for 2 hours at 37°C; then, 100 nM Dox or 10 μg/mL TZM was added to the conditioned media, and vessels were incubated overnight (15–20 hours; Dox) or for 2 nights (39–44 hours; TZM) prior to assessing vasomotor function.

### Statistics

Statistical analyses were performed in GraphPad Prism 9 with significance assessed as *P* < 0.05 unless otherwise noted. Details are provided in Supplemental Methods.

### Study approval

All study procedures conform to the Declaration of Helsinki and were approved by the IRB at Medical College of Wisconsin (protocols PRO00030688, PRO00028824, PRO00041443, and PRO00042653), where studies were conducted. All longitudinal study participants provided written, informed consent prior to data collection.

### Data availability

RNA-Seq data are available via the GEO repository (accession no. GSE261326), and all other deidentified data are available upon request. Values for all data points in graphs are reported in the Supporting Data Values file.

## Author contributions

JDT contributed to study conceptualization, conducting experiments, analyzing data, creating figures, writing the initial draft, and reviewing and editing the manuscript. LENT contributed to study conceptualization, project administration, conducting experiments, analyzing data, creating figures, writing the initial draft, and reviewing and editing the manuscript. SNH contributed to study conceptualization, conducting experiments, analyzing data, creating figures, and reviewing the manuscript. STH contributed to creating figures and reviewing and editing the manuscript. JCH and JL contributed to conducting experiments and analyzing data. IYV contributed to analyzing data and creating databases. ALK contributed to study conceptualization, project administration, and conducting experiments. AJK contributed to study conceptualization, conducting experiments, analyzing data, writing the initial draft, and reviewing and editing the manuscript. ZL contributed to conducting experiments, analyzing data, creating figures, writing the initial draft, and reviewing and editing the manuscript. RMK contributed to study conceptualization, methodology, funding acquisition, and reviewing and editing the manuscript. GM contributed to study conceptualization, analyzing data, and reviewing and editing the manuscript. DDG contributed to study conceptualization, methodology, funding acquisition, project administration, supervision, and reviewing and editing the manuscript. AMB contributed to study conceptualization, methodology, funding acquisition, project administration, supervision, creating figures, and reviewing and editing the manuscript.

## Funding support

This work is the result of NIH funding, in whole or in part, and is subject to the NIH Public Access Policy. Through acceptance of this federal funding, the NIH has been given a right to make the work publicly available in PubMed Central.

- NIH grant R01 HL133029, HL173549 (AMB)
- NIH grant T32 HL134643 (JDT and STH)
- American Heart Association grant SFRN847970 (AMB, AJK, and DDG)
- We Care Foundation Grant (AMB and ALK)
- Medical College of Wisconsin Cardiovascular Center Pre-PPG Grant (AMB)
- Advancing a Healthier Wisconsin – Redox Biology Grant (AMB)
- Jenny and Antti Wihuri Foundation (RMK)

## Supplementary Material

Supplemental data

ICMJE disclosure forms

Unedited blot and gel images

Supporting data values

## Figures and Tables

**Table 1:**
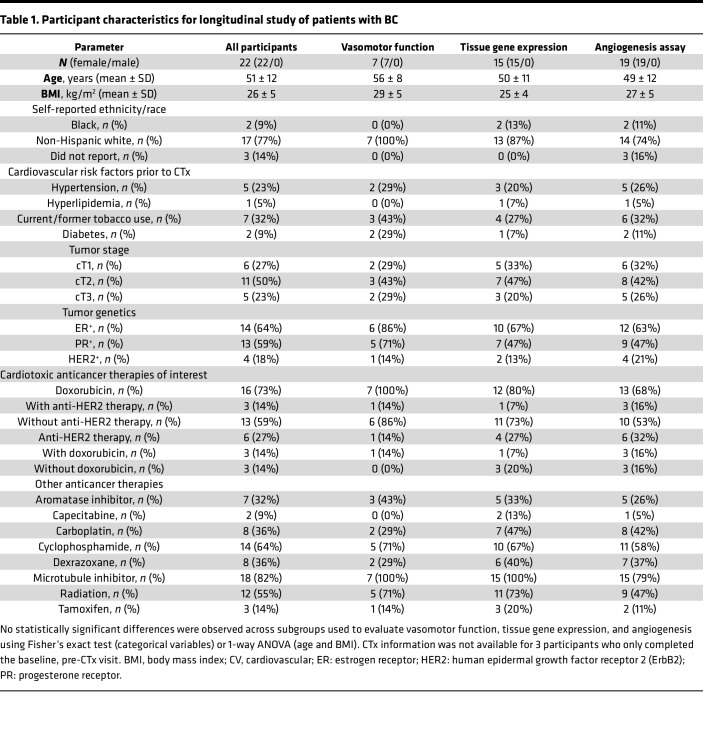
Participant characteristics for longitudinal study of patients with BC

## Version 1 09/30/2025

In-Press Preview

## Version 2 11/24/2025

Electronic publication
