# Supplementary material for: Characterization of anticancer therapy–induced microvascular dysfunction in patients with breast cancer supports targeted intervention
Source: JCI Insight. 2025 Sep 30;10(22):e194316. doi: 10.1172/jci.insight.194316 (PMC12643535; doi:10.1172/jci.insight.194316)
Supplement: Supplemental data [file jciinsight-10-194316-s261.pdf]

Supplementary Materials and Methods

Longitudinal study participants

Exclusion criteria included known metastatic disease, pregnancy, lactation, symptomatic hypotension or systolic blood pressure <100 mmHg, blood pressure >190/100 mmHg, eGFR <30 ml/min/1.73 m^2, hyperkalemia, active infection with COVID-19, HIV, hepatitis B or C, or MRSA/VRSA, contraindication or previous intolerance to ACE inhibitors, or a history of ischemic heart disease, myocardial infarction, cardiomyopathy, or heart failure. To better represent the broader population of breast cancer (BC) patients, we did not exclude patients on the basis of tobacco use or diagnoses of hypertension, hyperlipidemia, or diabetes.

Ex vivo exposure of adipose arterioles to anti-cancer therapeutic agents

Adipose arterioles from healthy donors were incubated overnight (15–24 h) at 37 °C, 5% CO_2 in Microvascular Endothelial Cell Growth Media (EGM-2, Lonza, CC-3202) containing doxorubicin (Dox, Sigma #D1515, 100 nM), trastuzumab (TZM, Selleckchem #A2007, 10 µg/ml), or paclitaxel (PTX, Sigma #T7191, 1 µM) prior to assessing vasomotor function. PTX was initially prepared in dimethyl sulfoxide (DMSO) and diluted in EGM-2. We have previously confirmed that the final dose of DMSO does not affect vasomotor function in isolated human arterioles (1). Pilot investigations suggested that the length of overnight exposure (i.e., 15 *vs.* 24 h) did not influence outcomes for vessels exposed to Dox or PTX, whereas longer exposure times seemed to correspond with greater vasomotor impairments in vessels exposed to TZM. Therefore, a second time point was added in which arterioles were incubated with TZM (10 µg/ml) for 48 h prior to analysis. We have previously confirmed that vasomotor function is preserved in control arterioles exposed to vehicle (EGM-2) for 48 h.

Vasomotor function and fluorescence microscopy

Adipose arterioles (diameter: 170 ± 5 µm) were suspended in oxygenated Kreb's buffer (37 °C, pH 7.4) within an organ chamber. Vessels were cannulated on glass micropipettes with matched impedance and pressurized to 60 mmHg via Kreb's-filled fluid reservoirs. Following an hour of equilibration, vessel diameter was measured with videomicroscopy, and endothelin-1 was added to the bath in incremental doses (final dose: 0.64 ± 0.04 nM) to pre-constrict vessels to 30–70% of baseline diameter. Vasoreactivity was then assessed in response to chemical and mechanical stimuli in separate trials. To evaluate flow-mediated dilation (FMD), fluid reservoirs were raised/lowered in equal and opposite directions to generate a pressure gradient and drive flow through the vessel lumen. Stepwise increases in pressure gradients were utilized to assess FMD across a range of flow stimuli, and diameter was measured after 5 min at each step. A dose-response curve was used to evaluate endothelium-dependent vasodilation to acetylcholine (ACh; 10^{-9}–10^{-4} M; 2 min per dose) added to the bath. Smooth muscle vasodilatory capacity (endothelium-independent) was evaluated in response to 100 µM papaverine. Experimental trials were separated by ≥30 min, and the chamber was washed three times with fresh Kreb's buffer between each trial.

Fluorescent imaging probes were utilized to quantify flow-induced microvascular production of nitric oxide (NO; ENZO Life Sciences #ENZ-51013-200) and mitochondrial hydrogen peroxide

(H_2O_2; MitoPY1, Tocris Bioscience #44-281-0, 10 μM). HEPES buffer containing the fluorescent probe was perfused through the lumen of cannulated vessels for 1 h prior to vessel pressurization, as described above. Following 1 h of equilibration, baseline images were captured using fluorescence microscopy (Olympus IX73 with C-cite FIRE LED lamp), then a shear stimulus was induced by generating a 100 cm H_2O gradient between the fluid reservoirs. Images were captured at 5 min (Supplemental Figure 1), and the percent change in fluorescence intensity from baseline (static) was calculated. Due to limited availability of vessels from the longitudinal study, immunofluorescence experiments were supplemented with cross-sectional samples obtained from breast cancer patients prior to or one month after CTx.

Angiogenesis assay

Freshly obtained adipose samples were embedded in Matrigel (Corning #354234) and incubated in Microvascular Endothelial Cell Growth Media (EGM-2, Lonza, CC-3202) at 37 °C, 5% CO_2 for two weeks to assess angiogenic potential based on a previously described method (2). Briefly, ~1 mm^3 adipose samples were embedded in 40 μl of Matrigel a 96-well cell culture plate on ice. Following 30 min incubation at 37 °C, 5% CO_2, 100 μl of warmed media (EGM-2, Lonza) ±CTx agents (500 nM Dox, 10 μg/ml TZM, or 1 μM PTX) was added to each well with eight replicates per condition. The plate was returned to the incubator, and media (±CTx agents) was changed twice weekly. Each well was imaged immediately after embedding (Day 0–1) and every 2–3 days afterward to observe capillary sprouting. The adipose sample and capillary sprouting area were traced in ImageJ, and the adipose area was subtracted from the capillary area to determine total sprouting area.

Transcriptomic analysis of primary endothelial cells

Endothelial cell preparation

Microvascular endothelial cells were isolated from adipose tissue from five donors without known cardiovascular disease using magnetic beads containing CD31 antibodies, as previously published (3). Donor characteristics are shown in the Supplemental Table 1. Endothelial cell identity was verified via FACs analysis with VE-cadherin to confirm that >85% of cells were endothelial cells. Cells from each donor were subcultured into five individual plates and grown on gelatin in EGM-2 (Lonza, CC-3202). Cells in a monolayer were treated with Dox (100 nM), TZM (10 μg/ml), PTX (1 μM), or their respective controls (media only for Dox and TZM; DMSO for PTX) for 24 hours with a total of five conditions per cultured endothelial cells from each donor.

Library preparation and RNASeq

RNA from cultured endothelial cells was prepared by phase separation using a TRIzol-chloroform method. RNA concentration and integrity were assessed using on a Fragment Analyzer (Agilent). Across all samples, the mean RNA integrity number (RIN) was 8.8 ± 0.19, and DV200 was 80.82% ± 1.88 (mean ± SEM). RNA libraries were prepared, and sequencing was conducted by the Mellowes Center for Genomic Sciences and Precision Medicine at the Medical College of Wisconsin (RRID:SCR_022926) in two batches, both following Illumina's TruSeq stranded mRNA library kit with unique dual indexing protocol.

Briefly, RNA was reverse transcribed into cDNA using random priming and PCR to add Illumina adaptors and barcodes. mRNA enrichment was conducted according to the kit protocol. Final libraries were amplified for sequencing with qPCR (Kapa Library Quantification Kit, Kapa Biosystems). Sequencing produced 150-bp paired end reads across up to two lanes on the Illumina NovaSeq6000 sequencer. Reads from samples run in multiple lanes were merged prior to quality control and trimming. Quality control and trimming of fastq files was done using Fastp (version 0.22.0) (4) using the default settings except for the minimum read length requirement setting, which was adjusted to 50bp. Average library size after filtering was 73.6 million reads per sample. The reads were aligned with STAR (version 2.7.10) (5), and count matrices were generated using htseq-count (version 0.13.5) (6), producing an average of 48 million assigned reads per sample. Samples were excluded from analysis if the percentage of uniquely mapped reads was <75% and/or >20% of reads mapped to multiple loci.

Differential gene expression analysis and gene set enrichment analysis

DESeq2 (version 1.42.1) was used to assess the differentially expressed genes within the Dox- or TZM-treated samples relative to untreated controls, while PTX-treated samples were compared to DMSO only (vehicle for PTX). Genes with adjusted p value < 0.05 and $\log_2$ fold change > 0.5 or < -0.5 were considered significantly differentially expressed. The model matrix accounted for unique patient ID as well as drug exposure conditions as fixed effects. All analyses were conducted in Rstudio (2023.06.2+562 "Mountain Hydrangea" Release). EdgeR (version 4.0.16) was also used to assess differential expression in an identical manner as described for DESeq2 (cutoff was adjusted p value < 0.05 and $\log_2$ fold change > 0.5 or < -0.5). GSEA (version 4.3.3) was used to perform gene set enrichment analysis for each condition and gene sets with false discovery rate (FDR) q value < 0.05 were considered significantly enriched.

Quantitative real-time polymerase chain reaction

Total mRNA was isolated from adipose tissue using the Qiagen RNeasy Lipid Tissue Mini Kit (#74804) and from microvessels using the New England Biolabs Monarch Total RNA Miniprep Kit (#T2010). cDNA was synthesized from 1 µg of RNA utilizing the Applied Biosystems High Capacity cDNA Reverse Transcription Kit (#4368814) and diluted to 10 ng per qPCR reaction. qPCR was utilized to evaluate gene expression levels using Qiagen QuantiTect Primer Assays for VEGF-A (Qiagen QuantiTect #QT01682072), VEGF-B (#QT00013783), VEGF receptor 1 (VEGFR1; #QT00073640), VEGF receptor 2 (VEGFR2; #QT00069818), Krüppel-like factor 2 (KLF2; #QT00204729), Krüppel-like factor 4 (KLF4; #QT00061033), platelet EC adhesion molecule 1 (PECAM-1; #QT00081172), Notch receptor 1 (NOTCH1; #QT01005109), matrix metalloproteinase 8 (MMP-8; #QT00029820), Aph-1 homolog A, γ secretase (APH1A; #QT00018291), angiopoietin 1 (ANGPT1; #QT00046865), vascular cell adhesion molecule 1 (VCAM-1; #QT00018347), NF-kappa-B inhibitor alpha (NFKBIA; #QT00014266), histidine triad nucleotide binding protein 2 (HINT2; #QT00203098), adenosine receptor A_{2a} (ADORA2A; #QT01184988), endothelial NO synthase (eNOS; #QT00089033), superoxide dismutase (SOD2), and major histocompatibility complex, class II, DM alpha (HLA-DMA1; #QT00197288) with SYBR green (Qiagen Quantinova SYBR Green PCR Kit #208054) in a BioRad CFX384 Real-Time PCR System. Data were visualized using BioRad CFX Manager.

Expression levels were normalized to 18S rRNA (#QT00199367) then normalized to the group average pre-CTx gene expression using the $2^{-\Delta\Delta Ct}$ method.

Statistics

Statistical analyses were performed with GraphPad Prism 9, and significance was assessed as $P < 0.05$ unless otherwise indicated (*e.g.,* RNA sequencing analyses). Data are shown as mean ± standard error except where noted. Multiple comparisons were performed using post hoc testing with Bonferroni's method when a significant main effect or interaction was observed.

Vasomotor responses of vessels exposed to CTx agents *ex vivo* were compared to control using two-way, repeated measures ANOVA (drug × vasodilator stimulus) with pressure gradient and ACh dose as repeated measures. Vasodilation to papaverine was compared to control using unpaired t-tests.

In the longitudinal study, vasomotor responses were compared using mixed model analyses with time point and vasodilator stimulus as fixed factors and subject as a random factor to account for missing time points. Flow-induced changes in NO and mitochondrial H_2O_2 production were compared using unpaired t-tests.

Sprouting area in the angiogenesis assay was compared using mixed model analyses with drug (control *vs.* CTx agent) or CTx time point (pre, mid, or post CTx) and time (days after plating) as fixed factors and subject as a random factor to account for missing time points. Total sprouting area on the final measurement day was compared using paired t-tests for *ex vivo* CTx exposure. As a result of COVID-19 protocols, the small sample size of adipose biopsies mid CTx limited statistical power to detect small effect sizes in angiogenic potential. Therefore, we utilized a t-test to evaluate the *a priori* hypothesis that angiogenesis is suppressed mid CTx compared to pre CTx.

In the cross-sectional study, vasodilation in response to flow and ACh were compared using two-way ANOVA with the shear stimulus or ACh dose as a repeated measure, and vasodilation to papaverine was compared using one-way ANOVA. A simple linear regression was utilized to evaluate the correlation between maximal flow-mediated dilation and time since CTx cessation.

Gene expression levels were compared using mixed model analyses with time point (pre, mid, or post CTx) as a fixed factor and subject as a random factor to account for missing time points. MMP-8 expression values were log transformed prior to analysis. Outliers were identified and removed from gene expression data using the modified Thompson Tau test prior to analysis.

References

1. Freed JK, Beyer AM, LoGiudice JA, Hockenberry JC, and Gutterman DD. Ceramide changes the mediator of flow-induced vasodilation from nitric oxide to hydrogen peroxide in the human microcirculation. *Circ Res.* 2014;115(5):525-32.
2. Rojas-Rodriguez R, Gealekman O, Kruse ME, Rosenthal B, Rao K, Min S, et al. Adipose tissue angiogenesis assay. *Methods Enzymol.* 2014;537:75-91.
3. Mammoto T, Torisawa YS, Muyleart M, Hendee K, Anugwom C, Gutterman D, et al. Effects of age-dependent changes in cell size on endothelial cell proliferation and senescence through YAP1. *Aging (Albany NY).* 2019;11(17):7051-69.
4. Chen S. Ultrafast one-pass FASTQ data preprocessing, quality control, and deduplication using fastp. *iMeta.* 2023;2(2):e107.
5. Dobin A, Davis CA, Schlesinger F, Drenkow J, Zaleski C, Jha S, et al. STAR: ultrafast universal RNA-seq aligner. *Bioinformatics.* 2013;29(1):15-21.
6. Anders S, Pyl PT, and Huber W. HTSeq--a Python framework to work with high-throughput sequencing data. *Bioinformatics.* 2015;31(2):166-9.

Supplemental Table 1. Tissue donor characteristics for cross-sectional study of breast cancer patients

Parameter	Total	CTx-naïve	1-mo post CTx	2–9-mos post CTx	12–24-mos post CTx
**N** (female/male)	36 (36/0)	11 (11/0)	12 (12/0)	8 (8/0)	5 (5/0)
**Age**, years (mean ± SD)	53 ± 14	56 ± 14	53 ± 15	53 ± 14	48 ± 10
**Self-reported ethnicity/race**					
Asian, n (%)	1 (3%)	0 (0%)	0 (0%)	1 (13%)	0 (0%)
Black, n (%)	1 (3%)	0 (0%)	1 (8%)	0 (0%)	0 (0%)
Hispanic, n (%)	1 (3%)	0 (0%)	1 (8%)	0 (0%)	0 (0%)
Non-Hispanic white, n (%)	29 (81%)	9 (82%)	9 (75%)	6 (75%)	5 (100%)
Did not report, n (%)	4 (11%)	2 (18%)	1 (8%)	1 (13%)	0 (0%)
**Cardiovascular risk factors**					
Hypertension, n (%)	2 (6%)	0 (0%)	1 (8%)	1 (13%)	0 (0%)
Hyperlipidemia, n (%)	2 (6%)	1 (9%)	0 (0%)	1 (13%)	0 (0%)
Coronary artery disease, n (%)	1 (3%)	0 (0%)	0 (0%)	1 (13%)	0 (0%)
Tobacco use, n (%)	3 (8%)	1 (9%)	0 (0%)	2 (25%)	0 (0%)
Congestive heart failure, n (%)	1 (3%)	0 (0%)	1 (8%)	0 (0%)	0 (0%)

No statistically significant differences were observed across groups using Fisher's exact test (categorical variables) or one-way ANOVA (age). CV, cardiovascular; ER: estrogen receptor; CTx, anti-cancer therapy.

Supplemental Table 2. Tissue donor characteristics for *ex vivo* incubation studies

Parameter	Total	Control	Dox	TZM	PTX	Dox + VEGF-B	TZM + VEGF-B
**N** (female/male)	62 (58/4)	15 (15/0)	9 (9/0)	11 (11/0)	10 (10/0)	8 (7/1)	9 (6/3)
**Age**, years (mean ± SD)	45 ± 13	43 ± 10	49 ± 15	41 ± 9	44 ± 10	46 ± 18	45 ± 16
**Self-reported ethnicity/race**							
Black, n (%)	10 (16%)	6 (40%)	1 (11%)	1 (9%)	2 (20%)	0 (0%)	0 (0%)
Hispanic, n (%)	5 (8%)	0 (0%)	2 (22%)	1 (9%)	1 (10%)	1 (13%)	0 (0%)
Non-Hispanic white, n (%)	45 (73%)	8 (53%)	6 (67%)	8 (73%)	7 (70%)	7 (88%)	9 (100%)
Did not report, n (%)	2 (3%)	1 (7%)	0 (0%)	1 (9%)	0 (0%)	0 (0%)	0 (0%)
**Cardiovascular risk factors**							
Hypertension, n (%)	7 (11%)	2 (13%)	2 (22%)	0 (0%)	0 (0%)	1 (13%)	2 (22%)
Hyperlipidemia, n (%)	3 (5%)	1 (7%)	0 (0%)	0 (0%)	0 (0%)	0 (0%)	2 (22%)
Tobacco use, n (%)	5 (8%)	1 (7%)	1 (11%)	1 (9%)	0 (0%)	1 (13%)	1 (11%)

No statistically significant differences were observed across groups using Fisher's exact test (categorical variables) or one-way ANOVA (age). Some donor tissues were used to investigate multiple conditions (e.g., Control and Dox) and/or parameters (e.g., responses to flow and acetylcholine); thus, n values vary between the table of donor characteristics compared to data shown in figures. CV, cardiovascular; Dox, doxorubicin; PTX, paclitaxel; TZM, trastuzumab; VEGF-B, vascular endothelial growth factor B.

Supplemental Table 3. Subject demographics and cell culture history for adipose-derived endothelial cells.

ID	Sex	Age	Race	BMI	Risk Factors	Culture Notes	Sequencing Results Excluded
1	F	38	White	21.3		1x Freeze/Thaw	None
2	F	38	White	20.8		1x Freeze/Thaw	None
3	M	49	Unknown	45.2		Used Fresh	None
4	M	39	White	25.0		Used Fresh	None
5	F	58	White	22.2	CRC, HTN	Used Fresh	TZM

None of the tissue donors had a history of cardiovascular disease. Subject #5 had a history of colorectal cancer (CRC) and hypertension (HTN). The sequencing results of endothelial cells treated with trastuzumab (TZM) from Subject #5 were excluded because they failed to meet the stringency requirement for mapped reads.

Supplemental Table 4. Overlap in differential gene expression analyses with DESeq2 and EdgeR

Condition	Overlap	DESeq2: unique	EdgeR: unique	DESeq2: total	EdgeR: total
Dox	844	159	127	1003	971
TZM	0	1	1	1	1
PTX	196	31	32	227	228

The total number of differentially expressed genes (DEGs) analyzed with DESeq2 and EdgeR is presented along with the number of overlapping DEGs between analyses and the number of DEGs unique to each analysis. Dox: doxorubicin, PTX: paclitaxel, TZM: trastuzumab

Supplemental Figures

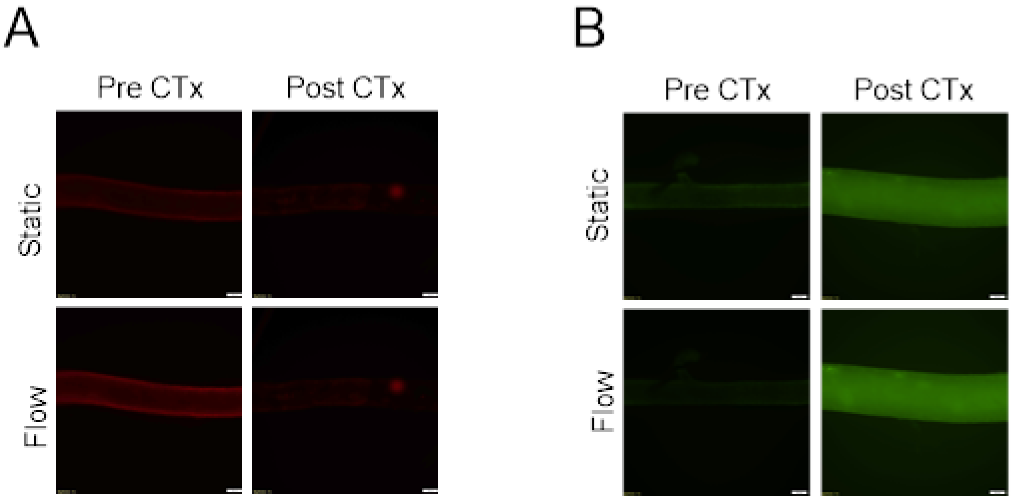

Supplemental Figure 1: Representative images of immunofluorescence studies. Fluorescent probes were utilized to quantify nitric oxide (NO) and mitochondrial hydrogen peroxide (H_2O_2) in adipose arterioles isolated from breast cancer patients before and after clinical anti-cancer therapy (CTx). Fluorescence for NO and H_2O_2 was quantified under static conditions and during a flow stimulus generated by a 100 cm H_2O pressure gradient. Representative images from one patient are shown in **A** and **B** for NO and H_2O_2, respectively.

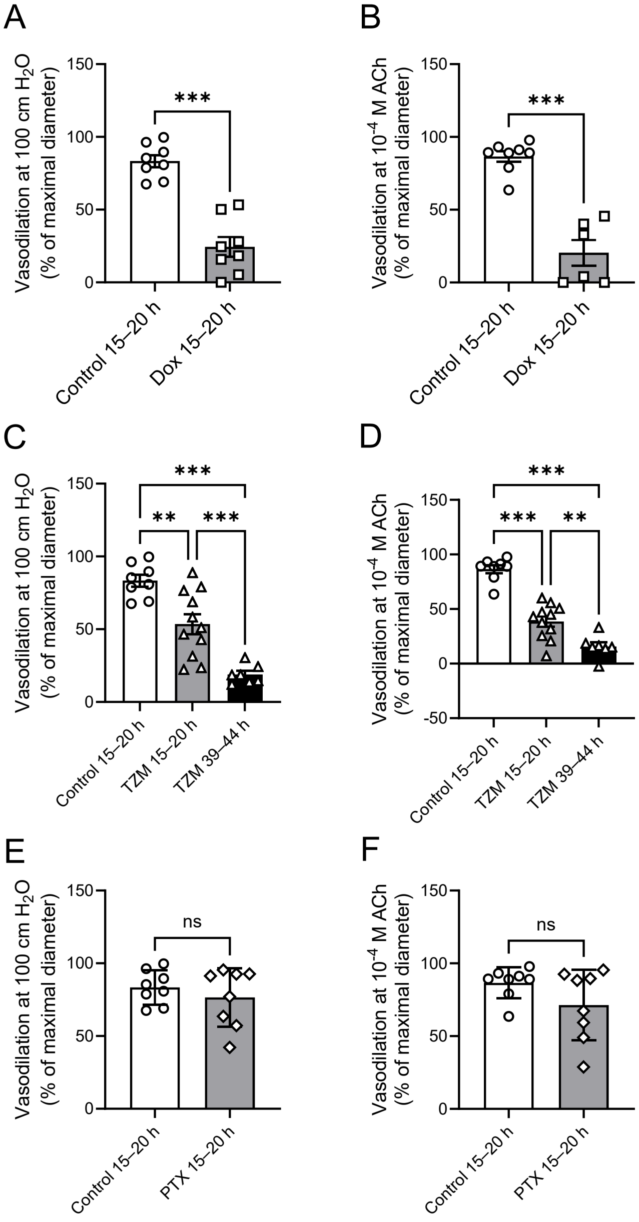

Supplemental Figure 2: Maximal dilation to flow and acetylcholine (ACh) in healthy arterioles incubated with anti-cancer therapies *ex vivo*. Endothelium-dependent vasodilation to flow generated by a pressure gradient of 100 cm H_2O and acetylcholine (ACh) at a dose of 10^{-4} M was impaired in healthy human adipose arterioles exposed to the cardiotoxic CTx agents doxorubicin (Dox) (**A** and **B**) or trastuzumab (TZM) (**C** and **D**) overnight (15–20 h). Endothelial function was further suppressed following two nights (39–44 h) of exposure to TZM (**C** and **D**). Maximal vasodilatory responses to flow and ACh were preserved in vessels exposed to paclitaxel (PTX) overnight (**E** and **F**). The same control data are plotted for each drug. $^{**}P < 0.01$, $^{***}P < 0.001$, unpaired t-test or mixed model analysis.

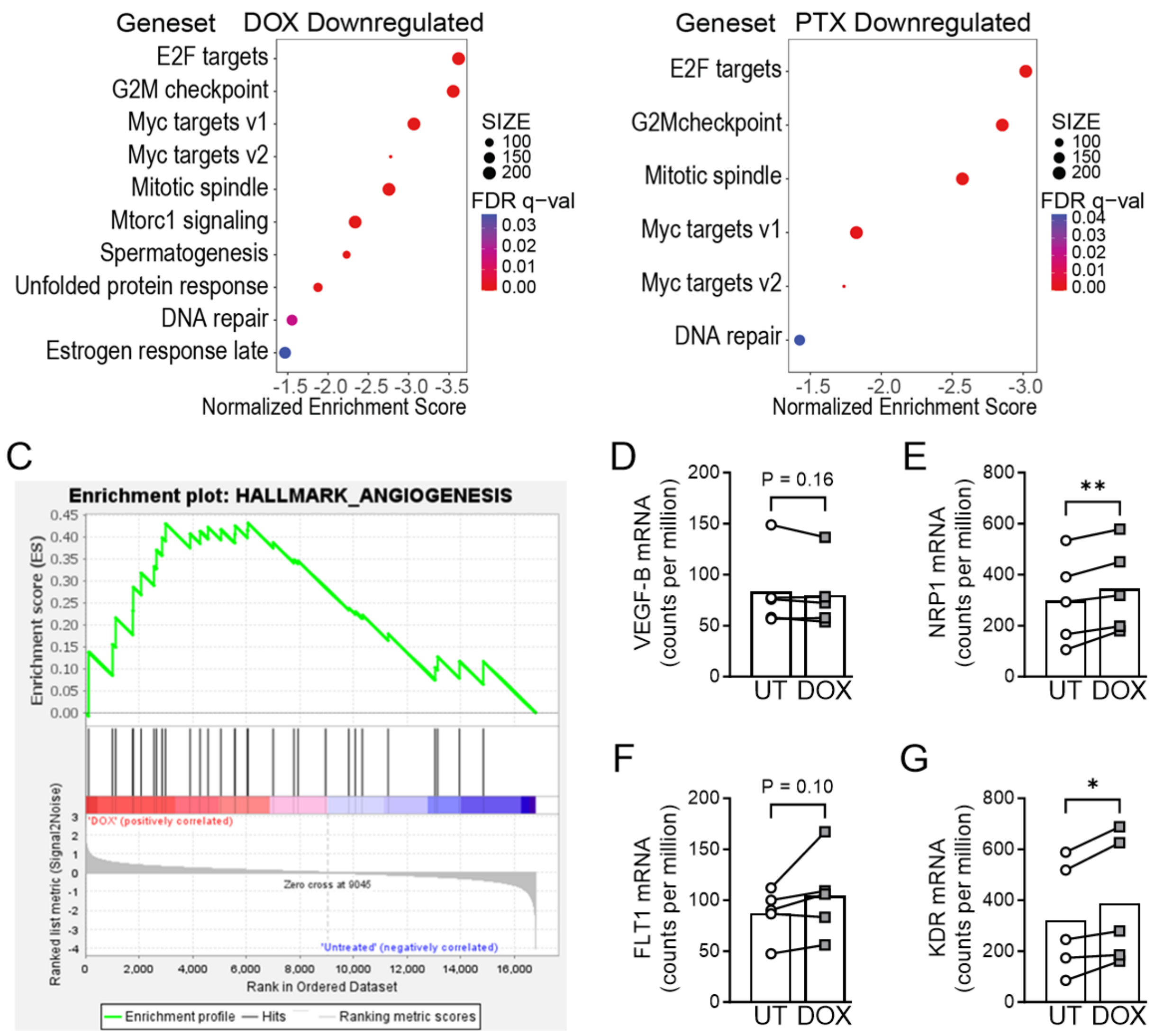

Supplemental Figure 3: GSEA analysis and mRNA expression of genes related to angiogenesis in endothelial cells treated with doxorubicin. Dot plots show gene sets that were downregulated in doxorubicin-treated (**A**, Dox, *n*=5) and paclitaxel treated (**B**, PTX, *n*=5) cells by GSEA analysis (FDR q-val < 0.05). Enrichment plot from GSEA shows the significant enrichment of the gene set "angiogenesis" in patient-derived endothelial cells exposed to Dox (**C**, related to Fig. 5D). mRNA expression of select genes related to vascular endothelial growth factor (VEGF) signaling in bulk RNAseq data was plotted as counts per million (**D–G**). Expression levels of VEGF-B (**D**), neuropilin receptor 1 (NRP1, **E**), VEGF receptor 1 (gene name FLT1, **F**), and VEGF receptor 2 (gene name KDR, **G**) in Dox-treated cells were compared to untreated cells. * $P < 0.05$; ** $P < 0.01$, paired two-tailed student's t-test.

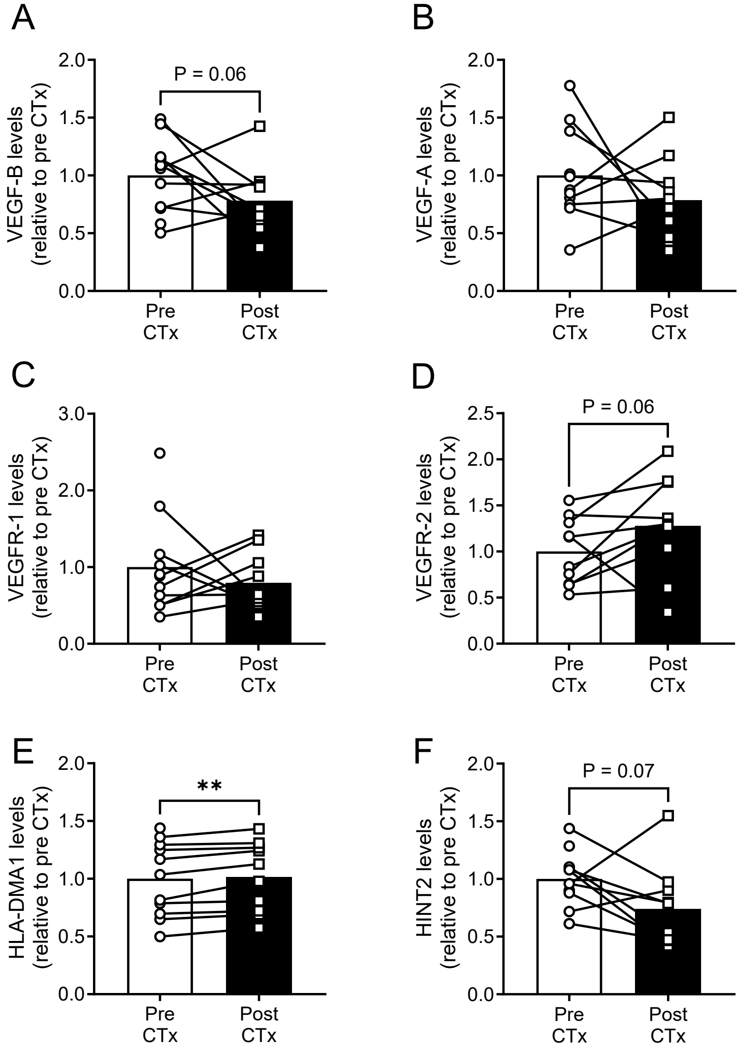

Supplemental Figure 4: Impact of anti-cancer therapy (CTx) on microvessel gene expression. Expression of genes related to angiogenesis, endothelial function, and inflammation is shown for microvessels obtained in a longitudinal study of breast cancer patients before and one month after CTx treatment. Ct values were normalized to 18s, then normalized to the group average pre-CTx value using the $2^{-\Delta\Delta Ct}$ method and compared using paired t-tests. ** $P < 0.001$. HINT2: histidine triad nucleotide binding protein 2, HLA-DMA1: major histocompatibility complex class II DM alpha chain, VEGF: vascular endothelial growth factor, VEGFR-1 and VEGFR-2: VEGF receptor 1 and 2.

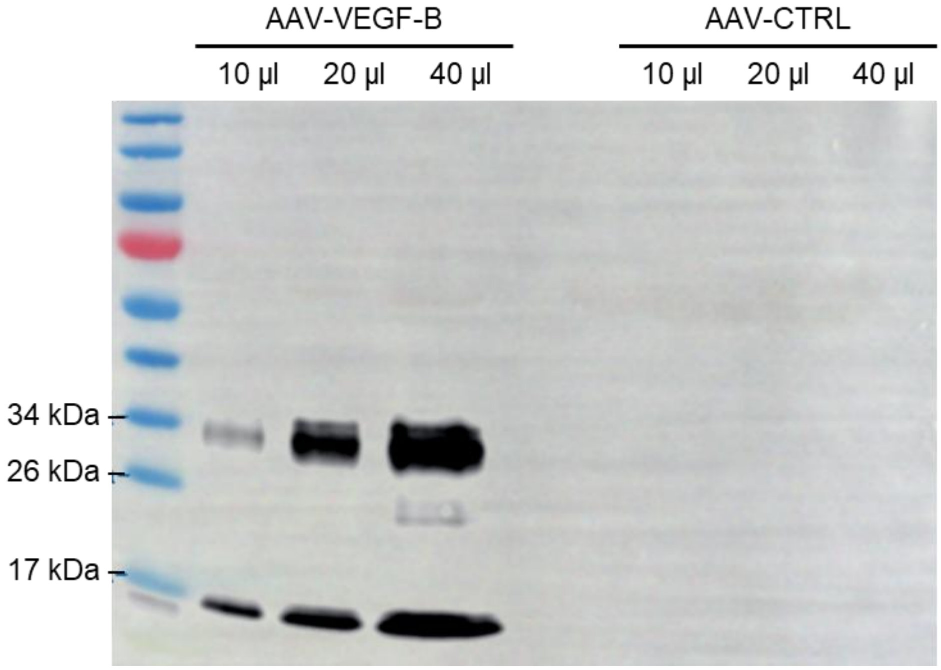

Supplemental Figure 5: Vascular endothelial growth factor B (VEGF-B) protein expression in conditioned media. A western blot of VEGF-B186 protein expression is shown for conditioned media collected from 293T cells transduced with an adeno-associated viral vector (AAV) expressing VEGF-B (VEGF-B-conditioned media) or containing a scrambled control sequence (CTRL; sham-conditioned media). 293T cells were washed to remove viral vectors prior to collecting conditioned media. Each well was loaded with 10–40 µl of conditioned media from 293T cells transduced with AAV-VEGF-B or AAV-CTRL.

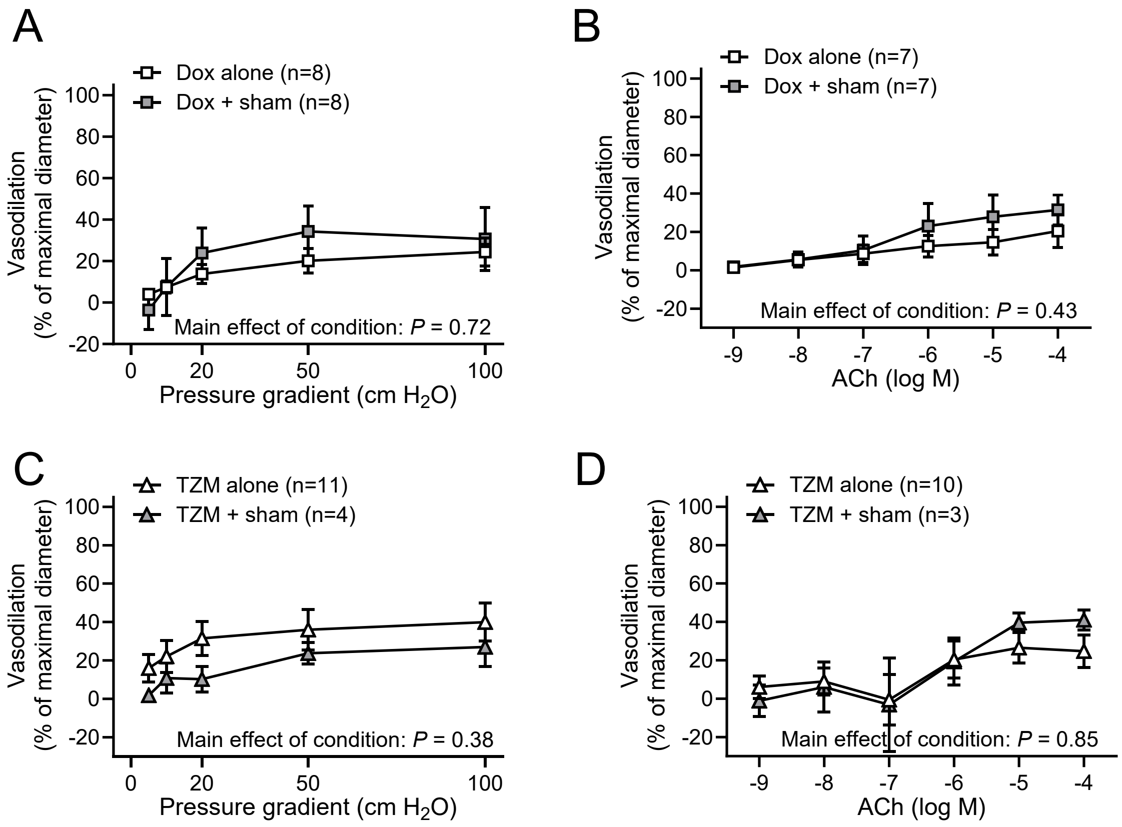

Supplemental Figure 6: Exposure to sham conditioned media does not alter the effects of doxorubicin or trastuzumab on microvascular endothelial function. In adipose arterioles from healthy human donors, exposure to sham conditioned media collected from 293T cells transduced with an adeno-associated viral vector containing a scrambled sequence did not alter the impact of *ex vivo* administration of doxorubicin (Dox, 100 nM, 15–24 h) or trastuzumab (TZM, 10 µg/ml, 48 h) on flow-mediated vasodilation (FMD) (**A** and **C**) or acetylcholine (ACh)-induced vasodilation (**B** and **D**) compared Dox or TZM alone (*i.e.,* in the absence of conditioned media). Data were compared via RM-ANOVA or mixed model analysis.
