# Supplementary material for: Characterization of anticancer therapy–induced microvascular dysfunction in patients with breast cancer supports targeted intervention
Source: JCI Insight. 2025 Sep 30;10(22):e194316. doi: 10.1172/jci.insight.194316 (PMC12643535; doi:10.1172/jci.insight.194316)

Image ID: 0000165_01
Acquire Time: 8.9.2016 10:37:53

Acquisition Information

#	Image ID	Acquire Time	Channels	Integration Times	Analysis	Image Name	Comment	Image Modifications
1	0000165_01	8.9.2016 10:37:53	Chemi 700	04:00 01:00	Manual	0000165_01		

Image Display Values

Channel	Color	Minimum	Maximum	K
Chemi	Gray Scale (Black on White)	0.00127	0.0734	0
700	Gray Scale (Black on White)	0.0339	0.141	0

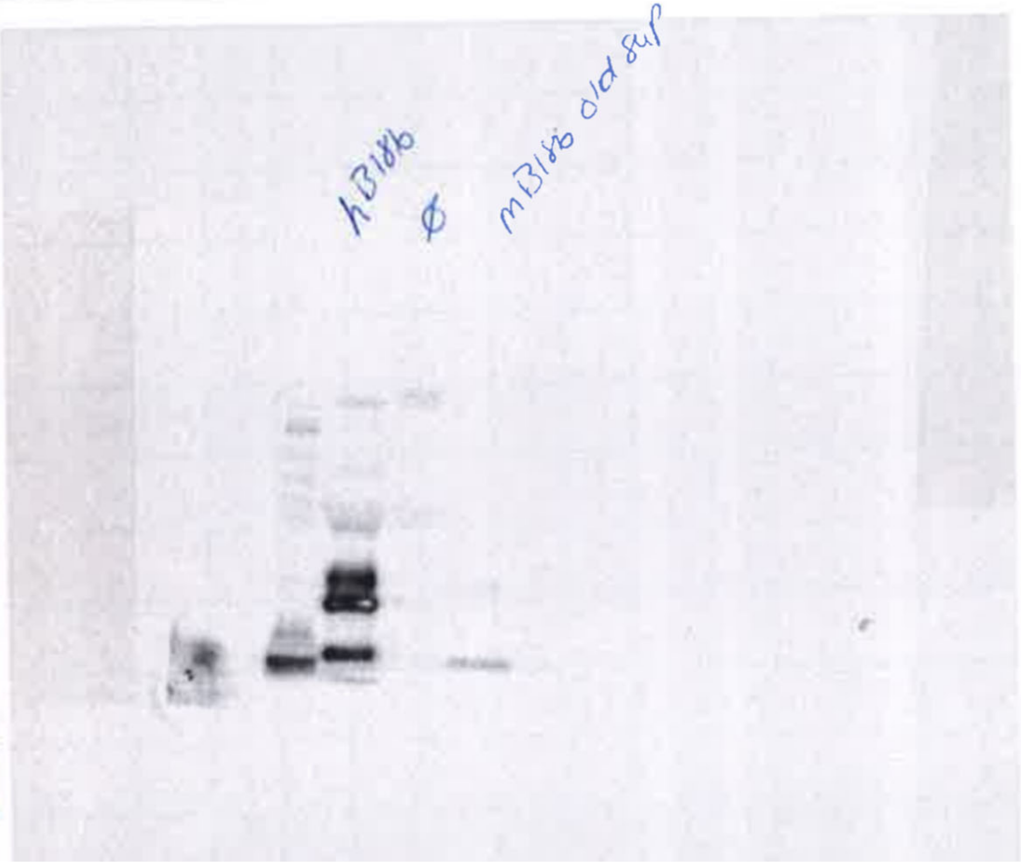

WB of 293T hB18b
expression into ECBM

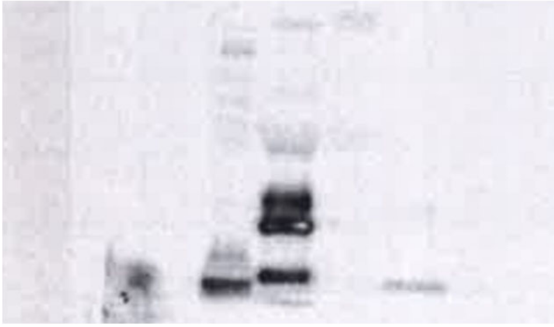

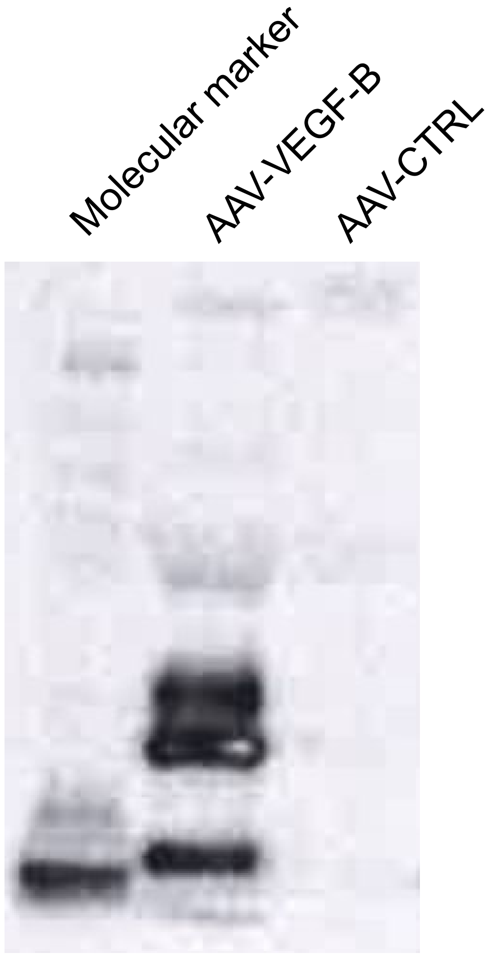

Supplemental Fig X. Western Blot of 297T transfected with hB186 (VEGFB expression vector) vs empty vector (Neg-CTRL) in ECBM

Image ID: 0000041_01

Acquire Time: Feb 16, 2024 1:30:30 PM

Page 1

Acquisition Information

#	Image ID	Acquire Time	Channels	Integration Times	Analysis	Image Name	Comment	Image Modifications
1	0000041_01	Feb 16, 2024 1:30:30 PM	R G B	Manual		24.02.06_12.17.54_PUB_600.tif		

Image Display Values

Channel	Color	Minimum	Maximum	K
R	Red	25.0	254	0
G	Green	23.0	254	0
B	Blue	17.0	254	0

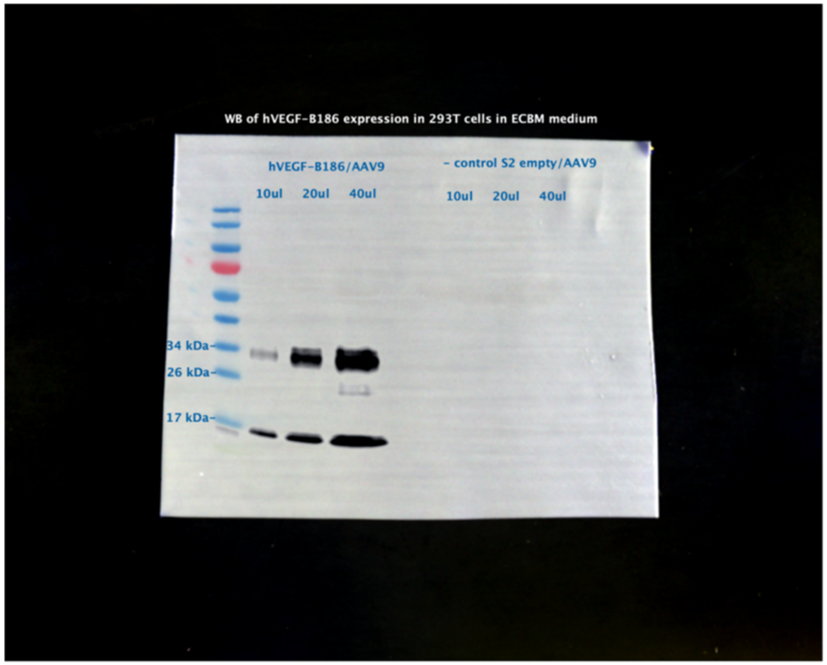

Supplement: Unedited blot and gel images [file jciinsight-10-194316-s263.pdf]
